# Chirality controlled responsive self-assembled nanotubes in water†
†Electronic supplementary information (ESI) available: Detailed experimental procedures and analyses, UV-vis absorption and CD spectroscopy, cryo-TEM and widefield microscopy. See DOI: 10.1039/c6sc02935c



**DOI:** 10.1039/c6sc02935c

**Published:** 2016-11-17

**Authors:** D. J. van Dijken, P. Štacko, M. C. A. Stuart, W. R. Browne, B. L. Feringa

**Affiliations:** a Centre for Systems Chemistry , Stratingh Institute for Chemistry , University of Groningen , Nijenborgh 4 , 9747 AG , Groningen , The Netherlands . Email: b.l.feringa@rug.nl; b Groningen Biomolecular Sciences and Biotechnology Institute , University of Groningen , Nijenborgh 7 , 9747 AG , Groningen , The Netherlands

## Abstract

The concept of using chirality to dictate dimensions and to store chiral information in self-assembled nanotubes in a fully controlled manner is presented. We report a photoresponsive amphiphile that co-assembles with its chiral counterpart to form nanotubes and demonstrate how chirality can be used to effect the formation of either micrometer long, achiral nanotubes or shorter (∼300 nm) chiral nanotubes that are bundled. The nature of these assemblies is studied using a variety of spectroscopic and microscopic techniques and it is shown that the tubes can be disassembled with light, thereby allowing the chiral information to be erased.

## Abstract 2

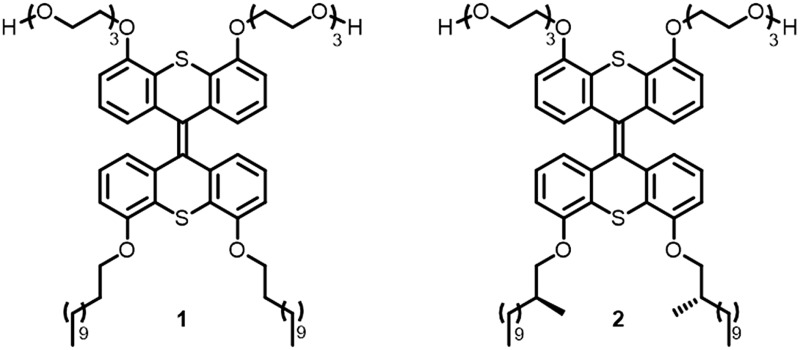
We report the design, synthesis and study of nanotube-forming light-responsive amphiphiles, in which chirality can be used as a means to control the morphologies of self-assembled structures.

## Introduction

A fascinating aspect and crucial feature of complex, multifunctional self-assembled objects found in nature is that they are dynamic, allowing a multitude of biological processes to rely on feedback-controlled communication.^1^ In other words, the self-assembled structure can adapt in response to an input signal. This ability to adjust in response to stimuli is crucial for the correct functioning of many bioprocesses.^2^ Therefore, the design and synthesis of artificial systems that can respond to external stimuli is highly warranted in order to better understand and mimic highly sophisticated natural systems. Furthermore, such systems potentially provide a basis for novel smart materials and nanoscale devices, for example for sensing and drug delivery.^3^

The use of chirality to control or initiate self-assembly offers a particularly interesting approach, as the living world around us is made up of chiral molecules of a unique handedness. Many natural systems, such as enzymes, are extremely effective in discriminating between enantiomers, and in the assembly of biomaterials like proteins and DNA, stereochemistry is a key factor, with a delicate interplay of molecular and supramolecular chirality.^4^ The amplification of chirality is necessary for the emergence of homochirality from a pool of nearly racemic compounds and is thought to be essential to the origin of life and the emergence of biomaterials and bionanosystems.^5^ In recent years, amplification of chirality has been exploited in the field of supramolecular chemistry and this principle has been applied to supramolecular polymers,^6^ gels,^7^ organic nanotubes,^8^ liquid crystals^9^ and other assemblies.^10^ The groups of Aida and Meijer in parallel pioneered the possibility to amplify the chirality in supramolecular systems, by exploring the “majority rules” principle,^8c,11^ whereby the major enantiomer determines the chirality of the entire assembly. In addition, the “sergeant–soldier” principle has been reported for the transfer of chirality from monomers to supramolecular aggregates.^12–14^ In the sergeant–soldier principle, as introduced by Green and coworkers,^15^ a minor amount of the chiral compound (sergeant), used as a dopant, dictates the overall chirality of a system that is made up of mainly achiral material (soldiers).

In natural phospholipids, the most abundant constituent of cell membranes, the chiral information is often present in the aliphatic tails, and the specific chiral information in these phospholipid backbones, present on the internal hydrophobic side of the self-assembled phospholipid bilayer, is of major importance.^16^ It is therefore remarkable that in most artificial systems, to the best of our knowledge,^8c,11–14,17^ the chiral information is introduced in the parts of the molecules that are not involved in the main binding interactions that lead to their assembly into a larger aggregate. Often, changes to these hydrophobic parts of the self-assembling molecules distort their packing to such an extent that well-defined supramolecular structures are no longer obtained.

## Results and discussion

### Design

**Fig. 1:**
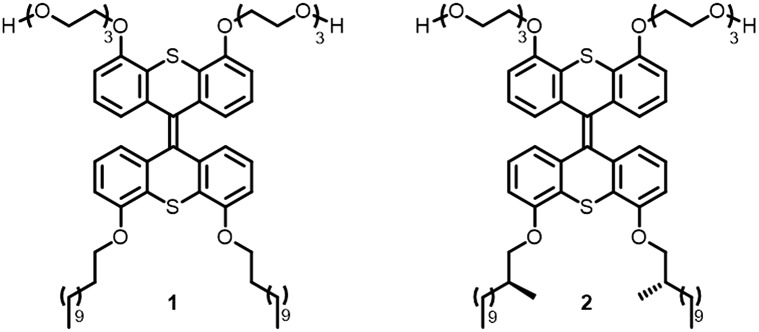
Design and structure of amphiphiles **1** (achiral) and **2** (chiral).

Inspired by nature, the present study focuses on the use of chirality as a tool to govern the defining parameters in complex supramolecular assemblies. We show that through molecular design, the properties of the well-defined aggregates as a whole, such as their dimensions, aggregation, overall chirality and photoresponsiveness, can be controlled. Previously, a light-responsive, self-assembled nanotube system based on a photochemically active amphiphile **1** was developed (Fig. 1).^18^ We envisioned that the amplification of chirality may be possible in such rigid supramolecular nanotubes by doping the achiral amphiphile **1** with small amounts of a closely related chiral analogue, and that we can use this stereochemical feature as a distinct control element for self-assembled nanotubes. In order to help achieve this goal, chiral **2** was designed, which bears two stereocenters in the hydrophobic part of the amphiphile. The structures of amphiphiles **1** and **2** contain a photosensitive overcrowded alkene unit that links two hydrophilic oligo-ethylene glycol headgroups with two hydrophobic alkyl tails. The bis-thioxanthylidene core provides a photoreactive and fluorescent functionality,^19^ and the oligo-ethylene glycol units facilitate its solubility in water. The hydrophobic alkyl chains are positioned at the core structure in order to maximise interaction and to facilitate supramolecular assembly by interdigitating upon aggregation to form a very robust bilayer, resulting in the formation of nanotubular assemblies.^18^

Here we show how the chirality and morphology of self-assembled supramolecular nanotubes are controlled by changing the molar fraction of the chiral constituent in the co-assembled nanotubes. The well-defined nanotubes have different diameters and show different behaviours depending on the ratio of chiral to achiral amphiphile building blocks. The chiral information, displayed by the nanotubes, can be erased with light *via* disassembly of the nanotubes. The nanotube systems presented here have several distinct differences in comparison with a number of supramolecular materials reported to date.^6–10^ The nanotubes are remarkably rigid, self-assemble in water and are photoresponsive, which means that they can be disassembled with light.^18^ Furthermore, the fact that our nanotubes form in water provides future opportunities for their biocompatibility.

As the hydrophobic half of the molecule makes up the internal part of the bilayer after self-assembly, and is most probably densely packed,^18^ we envisioned that the largest effect on the overall chirality could be obtained by introducing chirality in the hydrophobic alkyl chains. Furthermore, we reasoned that the packing of the molecules upon self-assembly would be least disturbed if the chirality were to be installed close to the aromatic core of the amphiphile. Two methyl groups were therefore introduced in amphiphile **2** at the C2 position of the alkyl chains (Fig. 1) to generate single enantiomers with the stereogenic information.

### Synthesis

**Scheme 1:**
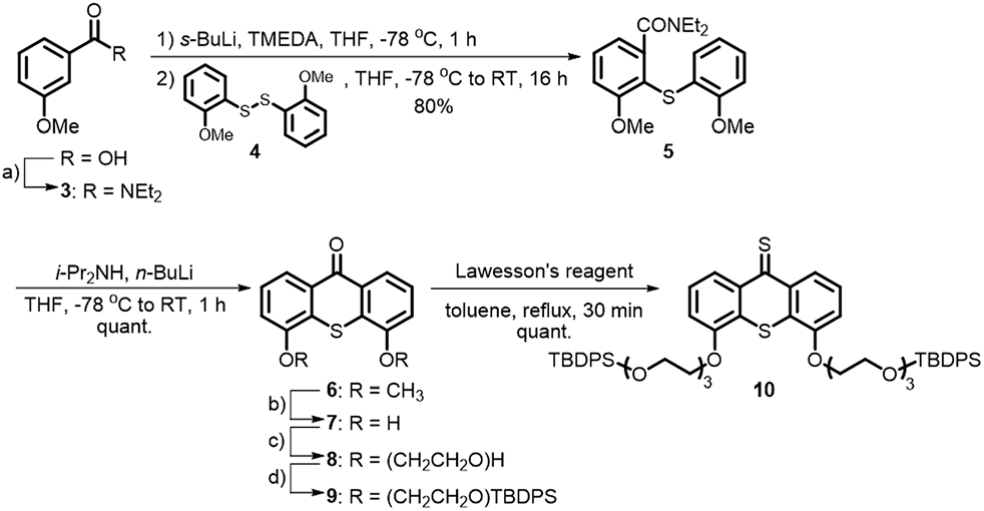
Synthesis of the hydrophilic precursor thioketone **10**. Reagents and conditions: (a) (1) SOCl_2_ (2.4 equiv.), CH_2_Cl_2_, reflux, 1 h; (2) Et_2_NH (4.0 equiv.), 0 °C to RT, 2 h, 98%; (b) BBr_3_ (5.0 equiv.), CH_2_Cl_2_, 0 °C to RT, 16 h, quant; (c) TsO(CH_2_CH_2_O)_3_H (2.0 equiv.), Cs_2_CO_3_ (5.0 equiv.), DMF, 110 °C, 16 h, 85%; (d) TBDPSCl (2.5 equiv.), imidazole (3.3 equiv.), CH_2_Cl_2_, 0 °C to RT, 1 h, 75%.

**Scheme 2:**
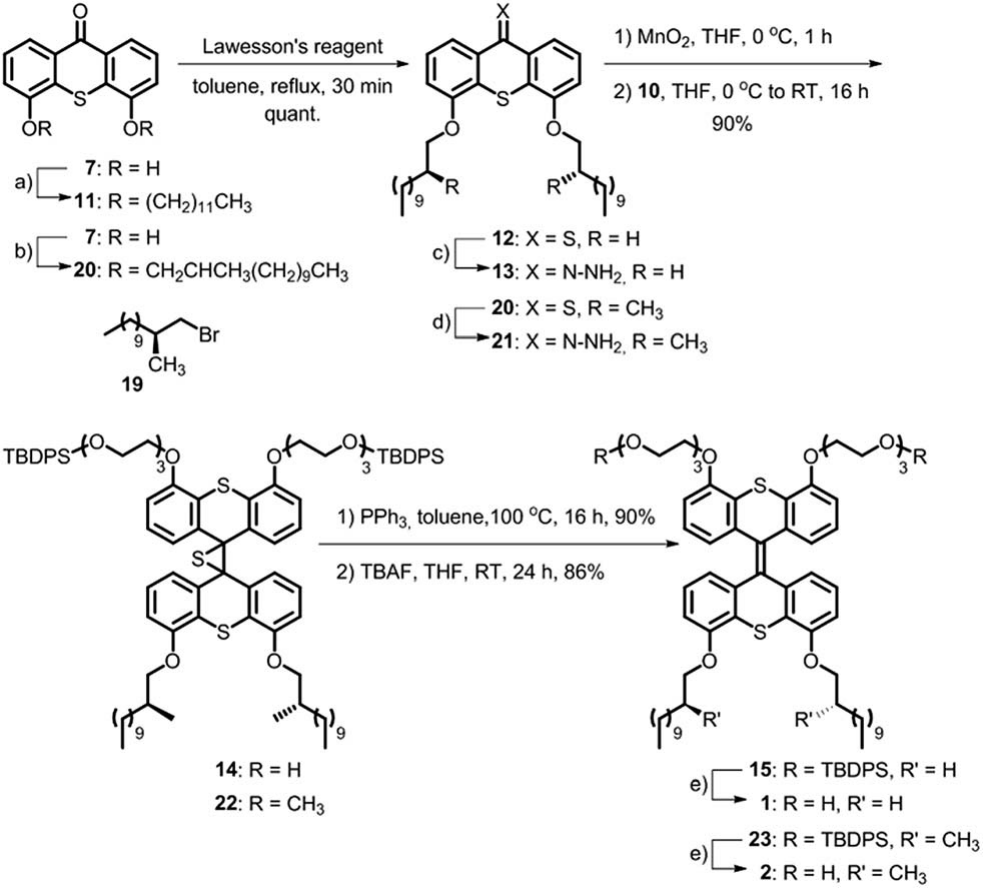
Synthesis of achiral amphiphile **1** and its chiral analogue **2**. Reagents and conditions: (a) CH_3_(CH_2_)_11_Br (2.6 equiv.), Cs_2_CO_3_ (5.0 equiv.), DMF, 110 °C, 16 h, 90%; (b) **19** (2.6 equiv.), K_2_CO_3_ (5.0 equiv.), DMF, 100 °C, 24 h, 91%; (c) NH_2_NH_2_·H_2_O (39 equiv.), THF, RT, 10 min, quant; (d) (1) Lawesson's reagent (1.5 equiv.), toluene, reflux, 2 h; (2) NH_2_NH_2_·H_2_O (19 equiv.), THF, RT, 10 min, 83%; (e) TBAF (2.8 equiv.), THF, 0 °C to RT, 24 h, 86–90%.

A major challenge in this work was the synthesis of both **1** and **2** in sufficient amounts to conduct the sergeant–soldier experiments. The syntheses of both amphiphiles were lengthy and contained some difficult steps. In this work, an efficient synthesis route to **1** and the chiral analogue **2** was developed (Schemes 1 and 2, see also ESI†).

Selective functionalisation of (thio)xanthones in the desired 4- and 5-positions is not a trivial task and the key building block **7** was therefore prepared using a bottom-up approach. 3-Methoxybenzoic acid was transformed into its acid chloride and quenched with diethylamine to give amide **3** in excellent yield (Scheme 1). Oxidation of 2-methoxybenzenethiol yielded disulfide **2**, which was added to **3** after selective *ortho*-lithiation with *s*-BuLi and TMEDA at –78 °C to give the thioxanthone precursor **5**. Regioselective lithiation using freshly prepared lithium diisopropylamine resulted in ring-closure to form **6**, which was subsequently deprotected with BBr_3_ to give the dihydroxy building block **7** with an overall yield of 78% over four linear steps. Hydrophilic ethylene glycol chains were introduced by deprotonation of the hydroxy moieties with Cs_2_CO_3_ and addition of an equimolar amount of mono-tosyl ethylene glycol. The terminal glycol groups of **8** were protected with TBDPS-groups and **9** was subsequently converted to its thioketone derivative **10**.

Hydrophobic chains were introduced by deprotonation of the hydroxy functionalities of **7** (Scheme 2) and addition of either dodecyl bromide or the chiral analogue **19** that was prepared using the Evans methodology according to the literature procedures.^20–23^ Only one diastereomer of the latest reported intermediate was observed (see ESI† for details) and it was therefore concluded that the bromide **19** is enantiomerically pure. Compounds **11** and **20** were converted to their thioketone analogues in order to increase their reactivities and subsequently converted to the hydrazones **13** and **21**, respectively, using hydrazine monohydrate at room temperature. The Barton–Kellogg reaction is routinely used for the construction of highly demanding tetra-substituted double bonds, but commonly suffers from low yields. The original synthesis of the amphiphile was considerably improved by employing a mild oxidation of the hydrazones using MnO_2_ at 0 °C, and subsequent addition of the thioketone **10** yielded the episulfides **14** and **22** in 90% yield. The desulfurisation of the episulfides was achieved with PPh_3_ and deprotection of the hydroxyl moieties with TBAF yielded the achiral amphiphile **1** and its chiral analogue **2** in 45% and 35% overall yields for the longest linear sequences (10 steps).

### Self-assembly

**Fig. 2:**
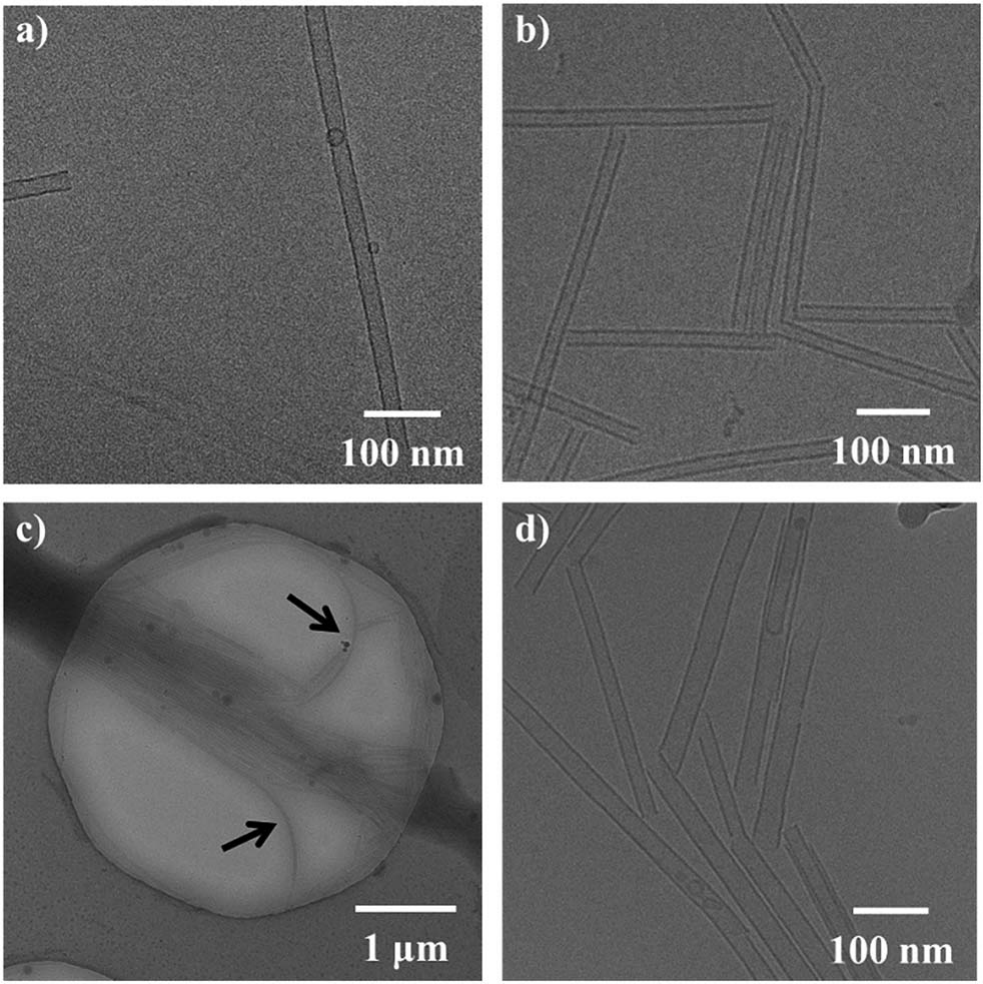
Cryo-TEM microscopy images of self-assembled nanotubes in water at a total concentration of 1 mg mL^–1^. (a) Nanotubes of achiral **1** with DOPC (1 : 1). (b) Nanotubes of chiral **2** with DOPC (1 : 1). (c) Nanotubes of **1** and **2** with DOPC (0.6 : 0.4 : 1). The arrows indicate the nanotubes bending away from the “bundle”. (d) Nanotubes of **1** and **2** with DOPC (0.4 : 0.6 : 1).

With achiral **1** and enantiopure **2** prepared, aggregation was first confirmed using cryo-TEM measurements. The amphiphile **1** self-assembles into micrometre long nanotubes (Fig. 2a) when co-assembled with 1,2-dioleoyl-*sn*-glycero-3-phosphocholine (DOPC) 1 : 1.^18^ Much to our delight, starting from a 1 : 1 mixture of **2** and DOPC, the formation of nanotubes from the chiral amphiphile was also observed (Fig. 2b). Henceforth, assembly experiments were always conducted with the amphiphile and DOPC in a ratio of 1 : 1 to the total amphiphile concentration. The nanotubes of **2** resemble the structures formed by the amphiphile **1**.

The bilayers making up the nanotube walls were found to be 3 nm thick for both the tubes of the pure achiral **1** and the homochiral amphiphile **2** and differences in the bilayers were not observed by cryo-TEM. No other types of aggregates were found for either **1** or **2**. Furthermore, the nanotubes of **1** were generally very straight, and virtually no bends or turns were observed in the nanotubes. Although we observed that for the nanotubes of **2** a higher amount of bent tubes was formed in comparison with the tubes of **1**, the nanotubes of **2** generally showed linear structures that were uniform in nature. A distinct difference between the nanotubes of pure achiral **1** and chiral **2** is that the nanotubes of **1** (Fig. 2a) were typically longer than a micrometre, while the tubes of **2** (Fig. 2b) were shorter, typically being only ∼300 nm long.

In addition, we found that the tubes of **2** tended to pack together more extensively (tube aggregation) than those of **1** at the same concentration, as observed by cryo-TEM. Fig. 2b and c for example, shows a type of network in which several nanotubes align and pack together to form “bundles” with a high content of (or exclusively) **2**. Nanotubes of achiral **1** were typically isolated and we found no evidence that they aligned with one another to a significant extent. While the reduced lengths of the nanotubes of **2** may be explained by a difference in the packing due to the presence of the two methyl moieties in the interacting hydrophobic tails (increase in hydrophobic volume), we do not currently know why the shorter tubes tend to bundle together. Nanotubes of both **1** and **2** capped with DOPC vesicles were also found, as reported for **1** previously,^18^ and the tubes were not different in this regard. After confirming that both the enantiopure amphiphile **2** and the achiral amphiphile **1** form nanotubes, we set out to perform sergeant–soldier experiments by mixing varying ratios of **1** and **2**. In the mixed nanotubes (Fig. 2c and d), nanotube formation was not inhibited at any ratio of the amphiphiles **1** : **2**. Increasing the fraction of chiral **2** to over 50% (Fig. 2d compared to Fig. 2c) resulted in tubes that were shorter than the nanotubes of the pure achiral **1**; these were typically ∼300 nm in length, and more bundled, which was reminiscent of the tubes of pure **2** (Fig. 2b). It should be noted that while in all the samples, both long and short tubes could be observed, at below 50% of **2**, the long tubes were far more abundant while the short tubes were mainly present in the mixtures containing over 50% of chiral **2**. Another observation is that the tubes of the mixed amphiphiles bend more, as can be seen in Fig. 2c (the nanotubes are bending away from the “bundle” as indicated by the black arrows).

### Spectroscopic studies

**Fig. 3:**
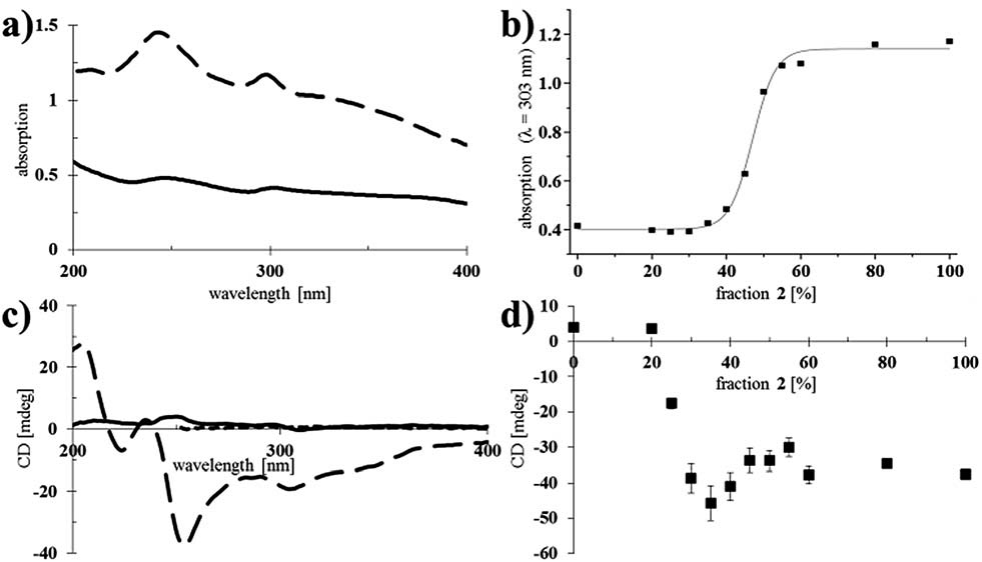
UV-vis absorption and CD spectra of nanotubes of **1** and **2**. (a) UV-vis absorption spectra of assemblies of pure **1** (solid line, 1.6 × 10^–4^ M in water) and pure **2** (dashed line, 1.6 × 10^–4^ M in water). (b) Absorption at *λ* = 303 nm as a function of the fraction of **2** in co-assembled nanotubes of **1** and **2** (data was fitted to a sigmoidal curve using Origin software). (c) CD spectra of nanotubes of **1** (solid line, 1.6 × 10^–4^ M in water) and **2** (dashed line, 1.6 × 10^–4^ M in water) and a solution of **2** (dotted line, 2.5 × 10^–5^ M in CHCl_3_; the cut-off for CHCl_3_ is *λ* = 260 nm). (d) CD maximum at *λ* = 303 nm as a function of the fraction of **2** in co-assembled nanotubes of **1** and **2**. The measurements were performed in triplicate and error bars are shown.

After having confirmed that nanotubes could be formed at different ratios of **1** and **2**, their spectroscopic properties were studied (Fig. 3).

Larger aggregates scatter more light and consequently, the baseline in the absorption spectra increases.^24^ Self-assembled samples of pure chiral **2** showed more scattering than those of pure achiral **1** (Fig. 3a), indicating the formation of relatively large bundles of nanotubes, which was consistent with the observations made by cryo-TEM (Fig. 2). Mixed nanotubes that were composed of <50% of the chiral amphiphile **2** showed similar absorption spectra to pure **1** (see ESI† for all UV-vis absorption spectra). Plotting the absorption (*λ* = 303 nm) as a function of the fraction of chiral amphiphile **2** in the mixed nanotubes, revealed a significant increase in scattering when the fraction of **2** exceeded 40%, with an undulation point at 47% (calculated by fitting a sigmoidal curve using Origin software; Fig. 3b). Interestingly, the absorption remained nearly unchanged when the fraction of chiral **2** was increased from 50% to 100% (Fig. 3b), which led us to propose that a homogeneous population of bundled, shorter nanotubes was formed when chiral **2** was the major component, while the formation of longer, isolated nanotubes was favoured when the tubes mainly consisted of achiral **1**. This is in agreement with the observation of more bundled, shorter nanotubes for the same samples, as measured by cryo-TEM (Fig. 2).

**Fig. 4:**
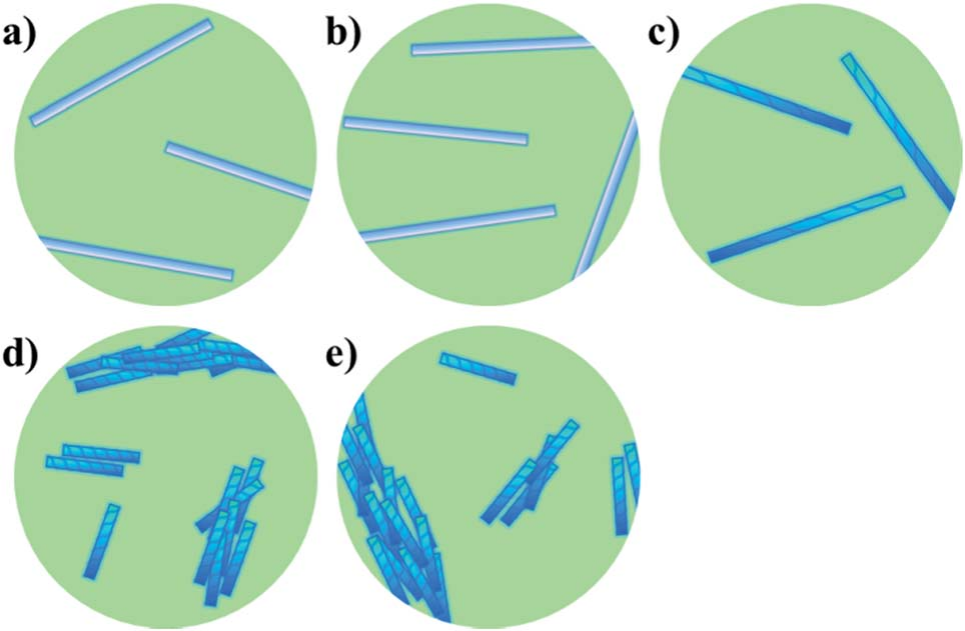
Schematic model for the observed behaviour of nanotubes consisting of achiral **1** and chiral **2** (with DOPC in a ratio of 1 : 1 amphiphile : DOPC) as a function of the amount of **2**. (a) Pure **1**; long, isolated achiral nanotubes. (b) <25% **2**; long, isolated achiral nanotubes. (c) 25–50% **2**; long, isolated chiral nanotubes. (d) >50% **2**; short, bundled chiral nanotubes. (e) Pure **2**; short, bundled chiral nanotubes.

We next set out to probe the induction of chirality based on the sergeant–soldier principle.^12–14,17^ We found that a solution of **2** in CHCl_3_ was CD silent (Fig. 3c, dotted line), likely because the chromophore is remote from, or not influenced by, the presence of the stereogenic centres. This offers good prospects for the concept of reading and erasing chiral information in the supramolecular assembly.^10a,25^ Nanotubes of achiral **1** were also CD silent, as expected (Fig. 3c, solid line). On the other hand, CD spectroscopy showed that nanotubes of the chiral amphiphile **2** in water exhibit cotton effects with negative maxima at *λ* = 303, 256 and 225 nm and a positive maximum at *λ* = 208 nm (Fig. 3c, dashed line). Subsequently, sergeant–soldier experiments were performed to investigate whether a small fraction of chiral **2** was able to induce chirality in the otherwise achiral nanotubes of **1**. Starting at 25% of **2** in the co-assembled aggregates, the mixed nanotubes were indeed chiral as shown by plotting the CD maximum (303 nm) as a function of the fraction of **2** (Fig. 3d). Nanotubes with less than 25% of **2**, however, did not show any CD signal, and plotting the CD maximum (*λ* = 303 nm) as a function of the fraction of chiral component **2** (Fig. 3d) revealed a similar relation as between the absorption and fraction of **2** (Fig. 3b). In contrast to the UV-vis absorption, which showed a sigmoidal relation with a sharp increase at 40% of **2**, the CD signal increased when the fraction of chiral compound **2** exceeded 25% and reached a maximum at 35%, after which it decreased and reached a constant value. Above a fraction of 50% of **2**, the nanotubes appeared to be homogeneous and no differences could be observed for tubes that consisted of 50–100% of the chiral amphiphile **2** (Fig. 3d). Fig. 4 shows a schematic model for the observed behaviour and properties of nanotubes consisting of achiral **1** and chiral **2** (with DOPC in a ratio of 1 : 1 amphiphile : DOPC) as a function of the fraction of **2**.

Cryo-TEM showed that the mixed nanotubes were micrometres long (Fig. 2) until **2** became the major component (>50%). It was hypothesised that mixtures of the amphiphiles containing less than 50% of **2** led to the formation of long, chiral nanotubes (Fig. 4c), which caused a sharp increase in the CD signal after exceeding a threshold of 20% of **2** (Fig. 3d). In the long nanotubes, the CD signal was more pronounced than in the short nanotubes.^26^ Apparently, the long nanotubes (larger aggregated structures) have a higher preference for the absorption of light of a particular handedness, compared to the shorter nanotubes, which led to a maximum value for the CD signal at 35% of **2**. At higher fractions of chiral **2**, an increase in absorption was observed, signifying the appearance of shorter, bundled tubes and a consequent decrease in CD, as in the short nanotubes, the chirality is less pronounced. When **2** was the major component (>50%) in the mixed tubes, both the UV-vis absorption (Fig. 3b) and the CD spectra (Fig. 3d) became nearly constant, indicating that further increasing the fraction of the chiral component **2** in the mixed nanotubes did not result in different self-assembled structures (Fig. 4d and e). These results show that chirality is a distinctive factor in controlling the length of individual self-assembled nanotubes, the aggregation of nanotubes and the chirality of the assembly.

**Fig. 5:**
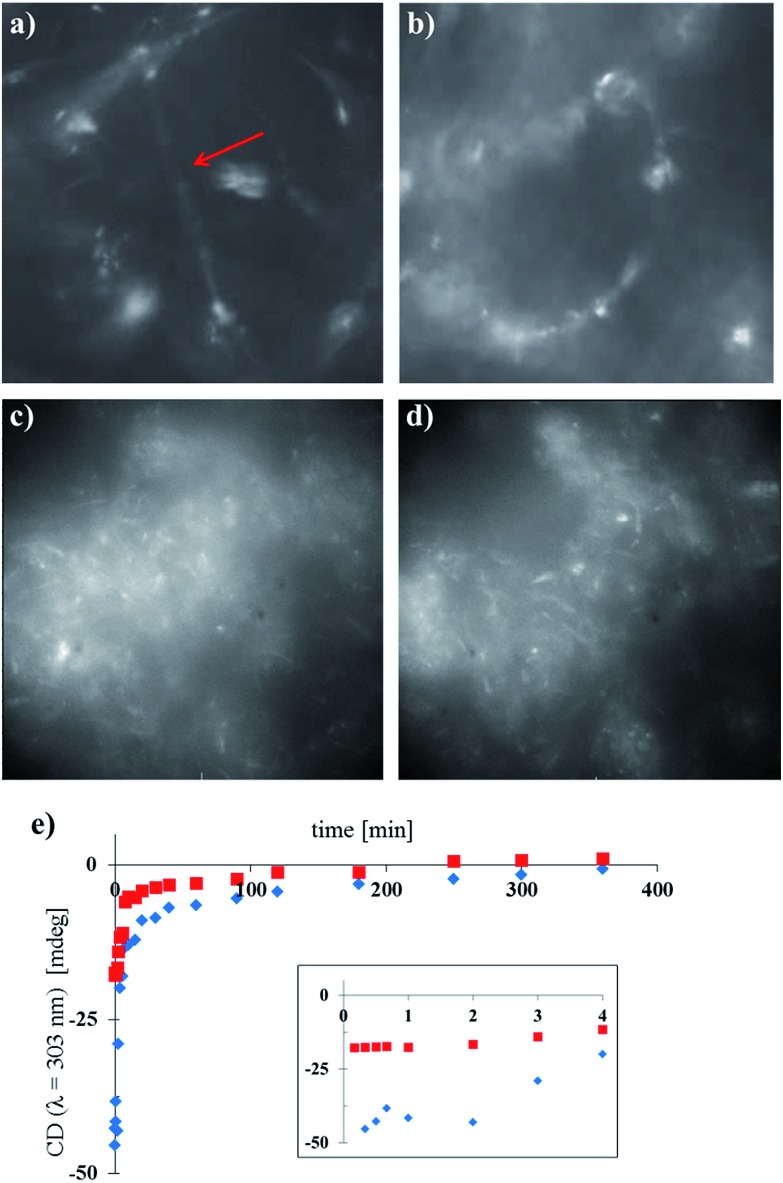
Photochemical disassembly of self-assembled nanotubes in water followed in real time by widefield fluorescence microscopy (a–d) and CD spectroscopy (e). (a) Image of pure **1** with DOPC (1 : 1) before irradiation. The isolated nanotube is indicated with a red arrow. (b) As in (a) after irradiation (*t* = 53 s, *λ*_irr_ = 390 nm). (c) Image of pure **2** with DOPC (1 : 1) before irradiation. (d) As in (c) after irradiation (*t* = 53 s, *λ*_irr_ = 390 nm). (e) Low intensity irradiation (*λ*_irr_ = 265 nm, 8 W) of long chiral nanotubes (**1** : **2** : DOPC 0.65 : 0.35 : 1, blue diamonds, 1.6 × 10^–4^ M) and short chiral nanotubes (**1** : **2** : DOPC 0.45 : 0.55 : 1, red squares, 1.6 × 10^–4^ M); inset is an expansion of *t* = 0–4 min.

The cores of the amphiphiles **1** and **2** are photoresponsive and can undergo cyclisation reactions induced by light (see ESI Fig. S19† for details) which affects the packing in the nanotubes. We were interested to see if the nanotubes of **1** and **2** and the mixtures of these compounds could be disassembled by light. In addition, as the nanotubes of **2** and the mixed nanotubes of **1** and **2**, where the fraction of **2** is higher than 25%, show significant CD signals, light-triggered disassembly of the chiral nanotubes would provide a way to erase the chiral information with light, offering intriguing possibilities for the development of soft memory devices.^27^ We followed the disassembly of the different nanotubes in real time, using widefield fluorescence microscopy and CD spectroscopy, showing the transition from nanotubes to less defined, larger aggregates (Fig. 5). As expected, long nanotubes were observed for samples of pure **1** (with DOPC 1 : 1, Fig. 5a), while the shorter nanotubes of pure **2** (with DOPC 1 : 1, Fig. 5c) showed large clusters of nanotubes in accordance with the observations by cryo-TEM (Fig. 2b). Irradiation *in situ* led to the deformation, and ultimately the disassembly, of the isolated nanotubes of pure **1** (Fig. 5b). Nanotube aggregates of pure **2** changed morphology under the same conditions as well (Fig. 5d), although visualisation of this process was complicated by their size and the resolution of the microscope. For the concomitant CD measurements (Fig. 5e), nanotube samples containing 35% and 55% of **2** were diluted and irradiated with low intensity UV light (*λ*_irr_ = 265 nm, 8 W, see ESI†). Note that the light intensity of the widefield microscopy setup was much higher, and the wavelength different, in comparison with the low intensity UV lamp, causing faster disassembly (Fig. 5a–d compared to Fig. 5e, further details in the ESI†). Both long and short chiral nanotubes showed a lag period for disassembly of 2 min under the given conditions. We hypothesise that upon initial irradiation, few molecules were cyclised and disassembly of the nanotubes initiated after a certain threshold of photochemically cyclised amphiphile was reached. After the lag period, the initial disassembly was relatively fast and slowed down over time. After approximately 2–3 h, the samples were CD silent and the chiral information in the system was erased due to the disassembly of the tubes.

## Conclusions

In summary, the design, synthesis and study of nanotube-forming light-responsive amphiphiles, in which chirality can be used as a means to control the morphologies of self-assembled structures, is presented. To the best of our knowledge, this comprises the first example of such nano-objects where the point chirality (stereogenic centre) is present in the hydrophobic part of the amphiphiles, much like in natural membranes. We hypothesise that the hydrophobic volume of the chiral amphiphile **2** is increased, compared to that of the achiral amphiphile **1**, due to the two methyl moieties in C2 of the hydrophobic tails. The increase in the hydrophobic volume distorts the long range packing of the amphiphile **2**. Unlike in other systems, however, the formation of nanotubes is not inhibited by the presence of the methyl moieties in the hydrophobic chains and rather causes the formation of shorter nanotubes.

Three distinct types of assemblies, namely (1) long, isolated achiral, (2) long, isolated chiral and (3) short, bundled chiral nanotubes, can be obtained and they were analysed using various spectroscopic and microscopic techniques. The ratio of chiral to achiral amphiphile provides a reliable handle to control the dimensions of the self-assembled nanotubes and, importantly, the chirality of the molecular constituent can be amplified to the aggregates as a whole. The fact that the nanotubes are in water and that the chirality, present in the hydrophobic part of the amphiphilic building blocks, is employed to control the morphology of the self-assembled nanotubes, takes the control over complexity of supramolecular systems one step further. In addition, the nanotubes can be disassembled with light and this responsive feature offers another control element towards smart materials.
