# Supplementary material for: Chirality controlled responsive self-assembled nanotubes in water
Source: Chem Sci. 2016 Nov 17;8(3):1783–9. doi: 10.1039/c6sc02935c (PMC5396556; doi:10.1039/c6sc02935c)
Supplement: Supplementary file 1 [file SC-008-C6SC02935C-s001.pdf]

Electronic Supplementary Material (ESI) for Chemical Science.
This journal is © The Royal Society of Chemistry 2016

Supporting information

Chirality Controlled Responsive Self-Assembled Nanotubes

D. J. van Dijken,[‡] P. Štacko,[‡] M. C. A. Stuart, W. R. Browne, B. L. Feringa[*]

‡ authors contributed equally

Contents

1. General 2
2. Synthesis 2
 2.1 Synthesis of achiral 1 2
 2.2 Synthesis of chiral 2 9
 2.3 NMR spectra of all new compounds 13
3. Self-assembly experiments 25
4. Cryo-transmission electron microscopy 25
 4.1 Enlarged cryo-TEM images 25
5. UV-vis absorption spectroscopy 29
6. Light-induced disassembly 30
 6.1 Widefield fluorescence microscopy 30
 6.2 CD spectroscopy 31
7. References 31

1. General

Starting materials and reagents were commercially available and used as obtained from Sigma-Aldrich or Acros without further purification. All solvents used for spectroscopic measurements, chromatography and the sergeant-soldier experiments were of analytical grade and degassed prior to use. Melting points were determined on a Büchi melting point B-545 apparatus. ^{1}H-NMR spectra were recorded on a Varian VXR-300 spectrometer (at 300 MHz) or a Varian MR400 (at 400 MHz) at ambient temperature. The splitting patterns are designated as follows: s (singlet); d (doublet); dd (double doublet); t (triplet); q (quartet); m (multiplet) and br (broad). ^{13}C-NMR spectra were recorded on a Varian VXR-300 spectrometer (at 75.4 MHz) or a Varian MR400 (100.6 MHz) at ambient temperature. Chemical shifts are denoted in δ (ppm), referenced to the residual protic solvent peak. Coupling constants *J*, are denoted in Hz. Masses were recorded with a Thermo scientific LTQ Orbitrap XL mass spectrometer. Silicycle Silicaflash P60, 40–63 m, (230–400 mesh) was used for column chromatography. Thin-layer chromatography (TLC) was performed on commercial Kieselgel 60F, 254 silica gel plates and compounds were visualized with UV light (λ = 254 nm) or $KMnO_4$ stain. Low intensity irradiations were performed with Spectroline ENB-280C/FE UV lamp. CD spectra were recorded on a JASCO J-715 spectropolarimeter and UV-vis measurements were performed on a Agilent 8453 UV-vis spectrophotometer. Weighing of small quantities was performed on a Mettler MT5 analytical microbalance.

2 Synthesis

We have optimized the synthesis of achiral **1** since the original published procedure by altering the reagents and conditions of the Barton-Kellogg coupling,[1] increasing the overall yield from 7% to 58% (for the longest linear sequence), starting from the same starting materials (Schemes S1-S4). Chiral analogue **2** was successfully synthesized in a similar way with an overall yield of 43% (for the longest linear sequence).

2.1 Synthesis of achiral 1

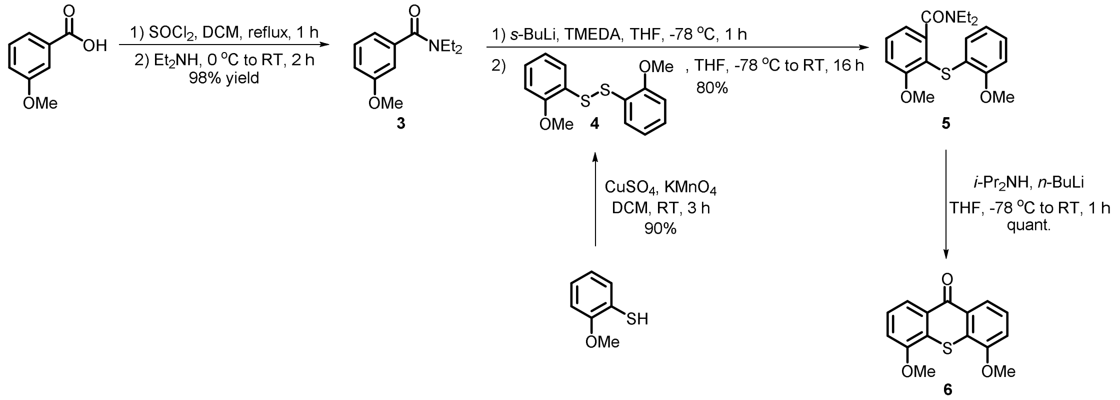

Scheme S1. Synthesis of key intermediate ketone **6** based on an adapted literature procedure.[2]

N,N-Diethyl-3-methoxybenzamide (3):

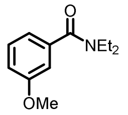

3-Methoxybenzoic acid (15.0 g, 98.6 mmol, 1.0 equiv) was dissolved in CH_2Cl_2 (100 mL) and $SOCl_2$ (27.3 mL, 237 mmol, 2.4 equiv) was slowly added. The mixture was heated at reflux for 1 h. After cooling down, the mixture was concentrated in vacuo and subsequently redissolved in CH_2Cl_2 (100 mL). The solution was slowly added to an ice bath cooled solution of Et_2NH (40.6 mL, 394 mmol, 4.0 equiv) in CH_2Cl_2 (100 mL) and stirred until all acid chloride had reacted (approximately 2 h). The mixture was then extracted with 1 M aq. HCl solution (2 × 100 mL) and subsequently with saturated aq. $NaHCO_3$ (2 × 100 mL). Evaporation of the solvent under reduced pressure gave **3** as a yellow oil (20.0 g, 96.6 mmol, 98%). ^{1}H NMR (300 MHz, $CDCl_3$) δ = 7.34 (d, *J* = 8.8 Hz, 1H), 6.96 – 7.00 (m, 3H), 3.87 (s, 3H), 3.85 (br s, 2H), 3.59 (br s, 2H), 1.29 (br s, 3H), 1.17 (br s, 3H) ppm; ^{13}C NMR (75.4 MHz, $CDCl_3$) δ = 170.5, 159.1, 138.1, 129.1, 117.9, 114.5, 111.3, 54.8, 42.9, 38.8, 13.9, 12.5 ppm; HRMS-EI *m/z* calculated for $C_{12}H_{17}NO_2$ $[M]^+$ 207.1259, found 207.1257.

1,2-Bis(2-methoxyphenyl)disulfane (4):

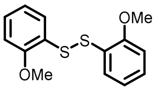

2-Methoxybenzenethiol (15.7 mL, 127 mmol, 1.0 equiv) was dissolved in CH_2Cl_2 (300 mL) and $KMnO_4$ (45.3 g, 286 mmol, 2.3 equiv) and $CuSO_4 \cdot 5\ H_2O$ (44.5 g, 178 mmol, 1.4 equiv) were added. The suspension was stirred at RT for 3 h. The mixture was filtered over celite and the cake washed with CH_2Cl_2 (100 mL). The filtrate was concentrated under vacuo and an off-white solid was obtained. After washing the solid with minimal amounts of CH_2Cl_2, disulfide **4** was obtained as a white solid (31.8 g, 114 mmol, 90%). m.p. 119.0 – 120.5 °C; ^{1}H NMR (300 MHz, $CDCl_3$) δ = 7.54 (dd, *J* = 7.7, 1.5 Hz, 1H), 7.16 – 7.22 (m, 1H), 6.88 – 6.94 (m, 1H), 6.85 (d, *J* = 8.1 Hz, 1H), 3.90 (s, 3H) ppm; ^{13}C NMR (75.4 MHz, $CDCl_3$) δ = 156.4, 137.6, 127.3, 124.2, 121.1, 110.3, 55.6 ppm; HRMS-EI *m/z* calculated for $C_{14}H_{14}O_2S_2$ $[M]^+$ 278.0435, found 278.0440.

N,N-Diethyl-3-methoxy-2-(2-methoxyphenylthio)benzamide (5):

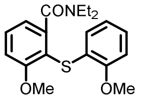

Dry THF (200 mL) was cooled to -80 °C under a nitrogen atmosphere. *s*-BuLi (18.5 mL, 21.2 mmol, 1.1 equiv) and TMEDA (3.20 mL, 21.2 mmol, 1.1 equiv) were subsequently added and the mixture stirred at -80 °C for 30 min. A solution of **3** (4.00 g, 19.3 mmol, 1.0 equiv) in dry THF (15 mL) was slowly added (5 min) and the mixture was stirred at -80 °C for 1 h. Compound **4** (6.50 g, 23.2 mmol, 1.2 equiv) was slowly added as a solid and the mixture was allowed to slowly warm to RT overnight. The mixture was diluted with Et_2O (200 mL) ,washed with 1 M aq. NaOH (200 mL) and concentrated in vacuo. The crude yellow solid was washed with a minimal amount of a mixture of *n*-pentane : EtOAc : CH_2Cl_2, 6 : 3 : 1 and the remaining off-white solid was purified by flash column chromatography (dry load, SiO_2, *n*-pentane : EtOAc : CH_2Cl_2, 6 : 3 : 1) to yield **5** as a white solid (5.30 g, 15.4 mmol, 80%). m.p. 140.9 – 142.6 °C; ^{1}H NMR (300 MHz, $CDCl_3$) δ = 7.41 – 7.46 (m, 1H), 7.02 – 7.07 (m, 1H), 6.93 – 6.95 (m, 2H), 6.80 (d, *J* = 8.4 Hz, 1H), 6.67 – 6.76 (m, 2H), 3.87 (s, 3H), 3.75 (s, 3H), 3.63 – 3.78 (m, 1H), 3.31 – 3.38 (m, 1H), 2.96 – 3.15 (m, 2H), 1.19 (t, *J* = 7.1 Hz, 3H), 0.98 (t, *J* = 7.1 Hz, 3H) ppm; ^{13}C NMR (75.4 MHz, $CDCl_3$) δ = 168.4, 160.3, 155.3, 144.8, 130.9, 126.5, 125.5, 125.1, 120.6, 118.3, 115.6, 111.0, 109.9, 55.8, 55.4, 42.4, 38.2, 13.6, 12.1 ppm; HRMS-EI *m/z* calculated for $C_{19}H_{23}NO_3S$ $[M]^+$ 345.1399, found 345.1390; elemental analysis calculated (%) for $C_{19}H_{23}NO_3S$: C, 66.10; H, 6.71; N, 4.05; S, 9.28; found (%): C, 66.00; H, 6.78; N, 4.04; S, 9.42.

4,5-Dimethoxy-9*H*-thioxanthen-9-one (6):

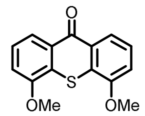

Compound **5** (15.0 g, 43.4 mmol, 1.0 equiv) was dissolved in dry THF (200 mL) under a nitrogen atmosphere and the solution added dropwise to a freshly prepared LDA solution (434 mL, 0.5 M, 5.0 equiv) cooled with an ice bath. The ice bath was removed and the mixture stirred at RT for 1 h. An 1 M aq. NH_4Cl solution (250 mL) was slowly added and the layers separated. The water layer was extracted with CH_2Cl_2 (2 × 200 mL) and the combined organic phase dried over Na_2SO_4 and concentrated in vacuo. The crude solid was purified by flash column chromatography (dry load, SiO_2, *n*-pentane : EtOAc, 2:1) to yield **6** as a yellow cotton-like solid (11.8 g, 43.4 mmol, quant.). m.p. 250 – 255 °C (dec); ^{1}H NMR (300 MHz, $CDCl_3$) δ = 8.25 (dd, *J* = 8.2, 0.9 Hz, 2H), 7.42 – 7.47 (m, 2H), 7.14 (d, *J* = 7.7 Hz, 2H), 4.05 (s, 6H) ppm; ^{13}C NMR (75.4 MHz, $CDCl_3$) δ = 180.2, 154.8, 129.9, 127.6, 125.9, 121.4, 112.1, 56.4 ppm; HRMS-EI *m*/*z* calculated for $C_{15}H_{12}O_3S$ $[M]^+$ 272.0507, found 272.0506; elemental analysis calculated (%) for $C_{15}H_{12}O_3S$: C, 66.20; H, 4.44; S, 12.44; found (%): C, 66.30; H, 4.41; S, 11.77.

*Note***:** LDA was prepared by adding *n*-BuLi (8.40 mL, 13.5 mmol, 5.0 equiv) to a solution of *i*-Pr_2NH (1.90 mL, 13.5 mmol, 5.0 equiv) in dry THF (65 mL) at -80 °C under a nitrogen atmosphere. The solution was allowed to warm to RT and stirred for 1 h.

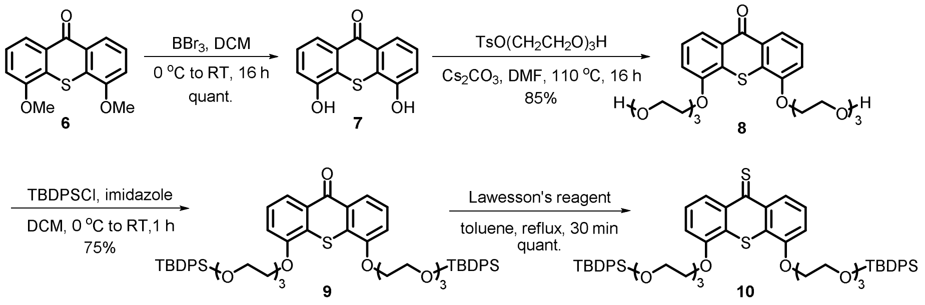

Scheme S2. Synthesis of the hydrophilic precursor, thioketone **10**, based on an adapted literature procedure.[1]

4,5-Dihydroxy-9*H*-thioxanthen-9-one (7):

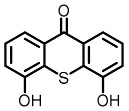

Compound **6** (2.0 g, 7.3 mmol, 1.0 equiv) was dissolved in dry CH_2Cl_2 (200 mL) under a nitrogen atmosphere and a 1 M boron tribromide solution (in CH_2Cl_2, 37 mL, 37 mmol, 5.0 equiv) was added dropwise at 0 °C. The mixture was allowed to warm to RT overnight. After cooling the mixture with an ice bath, water (150 mL) was slowly added and the layers separated. The organic layer was extracted with an 3 M aq. NaOH solution (3 × 150 mL). The water layer was subsequently acidified to pH = 1 with an 2 M aq. HCl solution and filtered through a glass filter. The residual cake was redissolved in acetone and concentrated in vacuo to yield **7** as a green solid (1.8 g, 7.3 mmol, quant.). m.p. 250 – 255 °C (dec); ^{1}H NMR (300 MHz, DMSO-d_6): δ = 10.98 (s, 2H, OH); 7.94 (d, *J* = 7.7 Hz, 2H); 7.38 (dd, *J* = 7.7, 7.7 Hz, 2H); 7.20 (d, *J* =7.7 Hz, 2H) ppm; ^{13}C NMR (50 MHz, DMSO-d_6): δ = 180.0, 153.7, 130.0, 127.0, 126.0, 119.9, 117.2 ppm; HRMS-APCI+ *m*/*z* calculated for $C_{13}H_9O_3S$ $[M + H]^+$ 245.0267, found 245.0259.

4,5-Bis(2-(2-(2-hydroxyethoxy)ethoxy)ethoxy)-9*H*-thioxanthen-9-one (8):

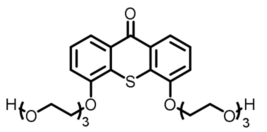

Compound **7** (5.80 g, 23.7 mmol, 1.0 equiv) was dissolved in DMF (150 mL). Cs_2CO_3 (16.5 g, 119 mmol, 5.0 equiv) and $TsO(CH_2CH_2O)_3H$ (14.1 g, 49.9 mmol, 2.0 equiv) were added and the mixture heated at 110 °C for 16 h. After cooling to room temperature, the reaction mixture was filtered over celite and the celite

cake washed with CH_2Cl_2. The solvents were removed by rotary evaporation and the residue purified by flash column chromatography (SiO_2, 5% MeOH in CH_2Cl_2) to yield **8** as a yellow solid (10.3 g, 20.1 mmol, 85%). m.p. 36 °C; ^{1}H NMR (400 MHz, $CDCl_3$): δ = 8.18 (dd, *J* = 8.2, 1.1 Hz, 2H), 7.36 (td, *J* = 8.1, 1.0 Hz, 2H), 7.10 (d, *J* = 8.0 Hz, 2H), 4.34 – 4.26 (t, *J* = 5.1 Hz, 4H), 3.95 (t, *J* = 5.1 Hz, 4H), 3.84 – 3.75 (m, 4H), 3.72 – 3.65 (m, 8H), 3.63 – 3.52 (m, 4H), 3.14 (s, 2H) ppm; ^{13}C NMR (100.6 MHz, $CDCl_3$): δ = 180.0, 153.9, 129.8, 127.9, 125.8, 121.6, 113.4, 72.6, 72.5, 71.0, 70.3, 70.1, 69.3, 69.1, 61.5 ppm; HRMS-APCI+ *m*/*z* calculated for $C_{25}H_{33}O_9S$ $[M + H]^+$ 509.1839, found 509.1818.

4,5-Bis(2,2-dimethyl-3,3-diphenyl-4,7,10-trioxa-3-siladodecan-12-yloxy)-9*H*-thioxanthen-9-one (9):

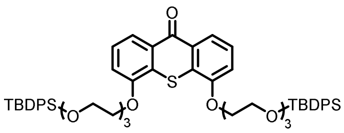

Compound **8** (7.60 g, 15.0 mmol, 1.0 equiv) was dissolved in CH_2Cl_2 (200 mL) and imidazole (3.40 g, 50.0 mmol, 3.3 equiv) was added at 0 °C. After 10 min, TBDPSCl (10.3 g, 37.4 mmol, 2.5 equiv) was added dropwise and after addition the ice bath was removed. The reaction mixture was stirred at RT for 1 h. The reaction mixture was filtered over celite and the solvent was removed by rotary evaporation. Compound **9** was obtained after flash column chromatography (SiO_2, *n*-pentane : EtOAc, 7 : 3) as a yellow, viscous oil (11.2 g, 11.3 mmol, 75%). ^{1}H NMR (300 MHz, $CDCl_3$): δ = 8.23 (d, *J* = 8.0 Hz, 2H), 7.76 – 7.62 (m, 10H), 7.43 – 7.29 (m, 10H), 7.13 (d, *J* = 7.9 Hz, 2H), 4.30 (t, *J* = 4.9 Hz, 4H), 3.95 (t, *J* = 4.9 Hz, 4H), 3.86 – 3.57 (m, 16H), 1.03 (s, 18H) ppm; ^{13}C NMR (75.4 MHz, $CDCl_3$): δ = 180.4, 154.4, 135.8, 133.9, 130.2, 129.9, 128.4, 127.9, 126.1, 121.9, 113.9, 72.7, 71.4, 71.1, 69.8, 69.5, 63.7, 27.1, 19.4 ppm; HRMS-ESI- *m*/*z* calculated for $C_{57}H_{68}O_9SSi_2Cl$ $[M + Cl]^-$ 1019.3805, found 1019.3780.

4,5-Bis(2,2-dimethyl-3,3-diphenyl-4,7,10-trioxa-3-siladodecan-12-yloxy)-9*H*-thioxanthene-9-thione (10):

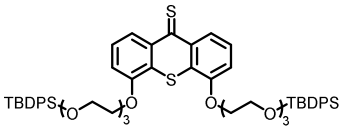

Compound **9** (4.0 g, 4.0 mmol, 1.0 equiv) was dissolved in dry toluene (100 mL) and Lawesson's reagent (5.0 g, 12 mmol, 3.0 equiv) was added. The reaction mixture was heated at reflux for 30 min. The solvent was removed by rotary evaporation and the crude material was purified by flash column chromatography (SiO_2, EtOAc : *n*-pentane, 2 : 1) to yield **10** as a viscous green oil (4.0 g, 4.0 mmol, quant.); ^{1}H NMR (400 MHz, $CDCl_3$): δ = 8.67 (d, *J* = 8.4 Hz, 2H), 7.70 – 7.66 (m, 10H), 7.42 – 7.31 (m, 14H), 7.14 (d, *J* = 7.9 Hz, 2H), 4.33 (t, *J* = 5.0 Hz, 4H), 3.97 (t, *J* = 5.0 Hz, 4H), 3.82 (t, *J* = 5.3 Hz, 4H), 3.77 – 3.73 (m, 4H), 3.72 – 3.65 (m, 4H), 3.62 (t, *J* = 5.3 Hz, 4H), 1.04 (s, 18H) ppm; ^{13}C NMR (100.6 MHz, $CDCl_3$) δ = 210.7, 154.2, 138.3, 135.6, 133.6, 129.6, 127.6, 126.3, 125.6, 112.8, 72.5, 71.2, 70.9, 69.5, 69.4, 63.4, 26.8, 19.2 ppm; HRMS-APCI+ *m*/*z* calculated for $C_{57}H_{69}O_8S_2Si_2$ $[M + H]^+$ 1001.3967, found 1001.3911.

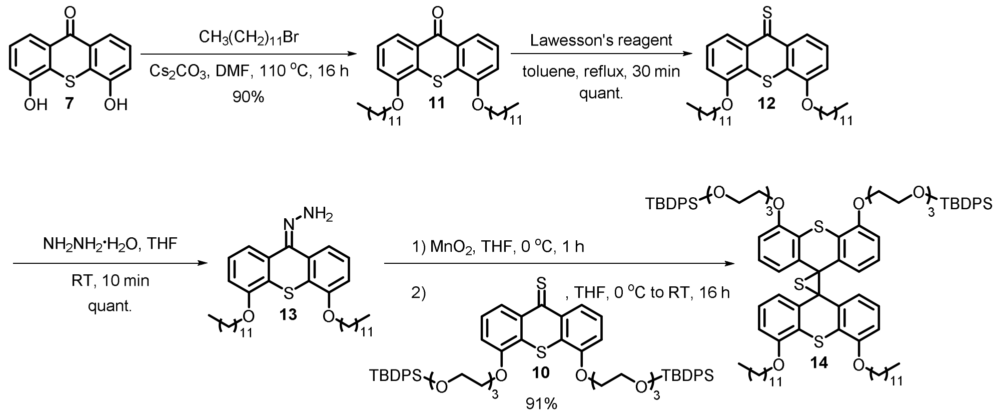

Scheme S3. Synthesis of the hydrophobic precursor, hydrazine **13** and coupling of **10** with **13** to give episulfide **14**, based on an adapted literature procedure.[1]

4,5-Bis(dodecyloxy)-9*H*-thioxanthen-9-one (11):

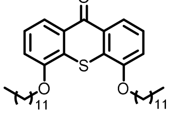

Compound **7** (0.50 mg, 2.0 mmol, 1.0 equiv) was dissolved in dry DMF (50 mL). Cs_2CO_3 (3.3 g, 10 mmol, 5.0 equiv) and dodecyl bromide (1.3 mL, 5.3 mmol, 2.6 equiv) were added and the mixture heated to 110 °C for 16 h. After cooling down to RT, the reaction mixture was filtered over celite and the solvent was removed by rotary evaporation. CH_2Cl_2 (50 mL) and H_2O (50 mL) were added and the layers separated. The water layer was extracted with CH_2Cl_2 (2 × 50 mL). The solvent was removed by rotary evaporation and the residue recrystallized from EtOH to yield **11** as a yellow solid (0.54 g, 1.8 mmol, 90%). m.p. 78 – 79 °C; ^{1}H NMR (400 MHz, $CDCl_3$): δ = 8.23 (dd, *J* = 8.1, 1.2 Hz, 2H), 7.41 (td, *J* = 8.1, 1.4 Hz, 2H), 7.12 (d, *J* = 8.0 Hz, 2H), 4.18 (t, *J* = 6.4 Hz, 4H), 2.00 – 1.85 (m, 4H), 1.65 – 1.55 (m, 4H), 1.44 – 1.20 (m, 16H), 0.87 (m, 6H) ppm; ^{13}C NMR (100.6 MHz, $CDCl_3$) δ = 180.4, 154.4, 130.0, 128.4, 125.8, 121.2, 113.0, 69.4, 31.9, 29.7, 29.6, 29.3, 29.0, 26.0, 22.7, 14.1 ppm; HRMS-APCI+ *m*/*z* calculated for $C_{37}H_{57}O_3S$ $[M + H]^+$ 581.4023, found 581.4002.

4,5-Bis(dodecyloxy)-9*H*-thioxanthen-9-thione (12):

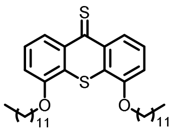

Compound **11** (2.0 g, 3.4 mmol, 1.0 equiv) was dissolved in toluene (100 mL) and Lawesson's reagent (4.17 g, 10.3 mmol, 3.0 equiv) was added. The reaction mixture was heated at reflux for 30 min. The solvent was subsequently removed by rotary evaporation. Purification by flash column chromatography (SiO_2, CH_2Cl_2 : *n*-pentane, 1 : 2) gave **12** as a green solid (2.0 g, 3.4 mmol, quant.). m.p. 78 – 79 °C; ^{1}H NMR (400 MHz, $CDCl_3$): δ = 8.66 (d, *J* = 8.5 Hz, 2H), 7.36 (t, *J* = 8.2 Hz, 2H), 7.10 (d, *J* = 7.8 Hz, 2H), 4.19 (t, *J* = 6.4 Hz, 4H), 2.02 – 1.86 (m, 4H), 1.67 – 1.54 (m, 4H), 1.48 – 1.15 (m, 32H), 0.87 (t, *J* = 6.7 Hz, 6H) ppm; ^{13}C NMR (100.6 MHz, $CDCl_3$): δ = 210.8, 154.5, 138.3, 126.3, 125.1, 124.0, 112.1, 69.6, 31.9, 29.7, 29.7, 29.4, 29.1, 26.1, 22.7, 14.1 ppm; HRMS-ESI+ *m*/*z* calculated for $C_{37}H_{57}O_2S_2$ $[M + H]^+$ 597.3794, found 597.3783.

(4,5-Bis(dodecyloxy)-9*H*-thioxanthen-9-ylidene)hydrazine (13):

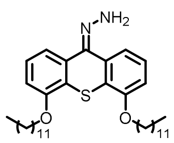

Compound **12** (2.14 g, 3.58 mmol, 1.0 equiv) was dissolved in THF (30 mL). The solution was treated at room temperature with 40% aq. hydrazine (8.00 mL, 140 mmol, 39 equiv). The solution decolorized to an orange solution within several min. The solvent and excess hydrazine were removed at reduced pressure. The residue was purified by flash column chromatography (SiO_2, *n*-pentane : EtOAc, 10 : 1) to yield **13** as a yellow solid (2.12 g, 3.56 mmol, quant.). m.p. 81 °C; ^{1}H NMR (300 MHz, $CDCl_3$): δ = 7.63 (d, *J* = 7.8 Hz, 1H), 7.43 (d, *J* = 7.8 Hz, 1H), 7.25 (m, 2H), 6.87 (d, *J* = 8.1 Hz, 1H), 6.82 (d, J = 7.9 Hz, 1H),

5.84 (s, 2H), 4.08 (dd, J = 14.2, 6.7 Hz, 4H), 1.88 (dd, J = 13.8, 6.9 Hz, 4H), 1.55 (m, 4H), 1.35 (m, 20H), 0.87 (t, J = 7.6 Hz, 6H) ppm; ^{13}C NMR (75.4 MHz, $CDCl_3$): δ = 155.9, 154.3, 142.0, 135.3, 127.0, 126.7, 125.7, 121.8, 120.1, 118.4, 111.2, 110.2, 69.4, 32.2, 29.9, 29.6, 29.4, 26.3, 22.9, 14.4 ppm; HRMS-APCI+ *m/z* calculated for $C_{37}H_{59}N_2O_2S$ $[M + H]^+$ 595.4292, found 595.4266.

Episulfide (14):

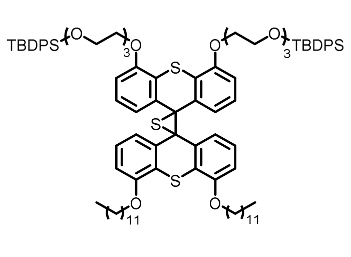

Solid MnO_2 (393 mg, 4.53 mmol, 10 equiv) was added to a solution of **13** (350 mg, 0.590 mmol, 1.3 equiv) in THF (50 mL) at 0 °C. The resulting solution was stirred for 1 h at 0 °C and filtered through a plug of silica gel. The plug was washed with a small amount of THF (10 mL). The light green solution was cooled to 0 °C. A solution of **10** (453 mg, 0.450 mmol, 1.0 equiv) in THF (2.4 mL) was added dropwise and the resulting mixture was stirred overnight. The solvents were evaporated under reduced pressure and the residue purified by flash column chromatography (SiO_2, *n*-pentane : EtOAc, 5 : 1) to yield **14** as a light yellow oil (638 mg, 0.410 mmol, 90%). ^{1}H NMR (400 MHz, $CDCl_3$) δ = 7.70 (d, J = 6.7 Hz, 10H), 7.40 – 7.33 (m, 12H), 7.32 (d, J = 7.9 Hz, 2H), 7.28 (d, J = 7.8 Hz, 2H), 6.85 (dd, J = 10.5, 5.4 Hz, 4H), 6.56 (d, J = 7.9 Hz, 2H), 6.51 (d, J = 8.0 Hz, 2H), 4.15 – 4.03 (m, 4H), 4.01 – 3.89 (m, 4H), 3.82 – 3.70 (m, 10H), 3.65 – 3.60 (m, 10H), 1.91 – 1.66 (m, 2H), 1.28 (m, 4H), 1.07 (s, 34H), 0.90 (t, J = 6.1 Hz, 6H) ppm; ^{13}C NMR (100.6 MHz, $CDCl_3$) δ = 154.1, 153.8, 135.8, 133.9, 132.6, 132.1, 129.8, 127.9, 126.0, 125.7, 125.2, 125.0, 124.0, 123.3, 120.1, 118.4, 111.6, 111.2, 110.3, 110.2, 72.7, 71.3, 71.0, 69.8, 69.5, 69.4, 63.6, 32.2, 30.0, 29.9, 29.8, 29.7, 29.6, 29.5, 29.4, 27.1, 26.3, 26.2, 23.0, 19.4, 14.4 ppm; HRMS-APCI+ *m/z* calculated for $C_{94}H_{123}O_{10}S_3Si_2$ $[M + H]^+$ 1564.7845, found 1564.7883.

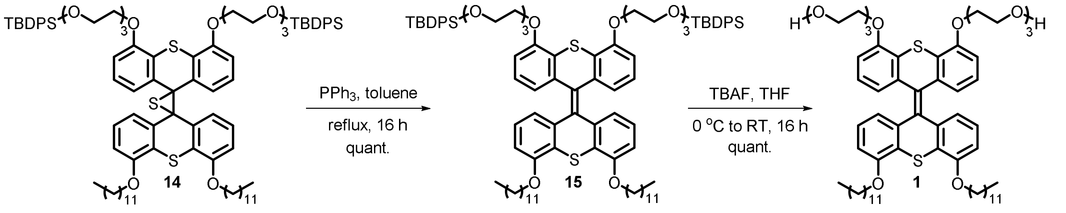

Scheme S4. Synthesis of achiral amphiphile **1** based on an adapted literature procedure.[1]

12,12'-((4',5'-Bis(dodecyloxy)-[9,9'-bithioxanthenylidene]-4,5-diyl)bis(oxy))bis(2,2-dimethyl-3,3-diphenyl-4,7,10-trioxa-3-siladodecane) (15):

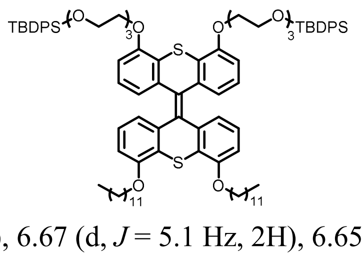

Compound **14** (3.6 g, 2.3 mmol, 1.0 equiv) was dissolved in toluene (140 mL) and PPh_3 (1.8 g, 6.9 mmol, 3.0 equiv) was added. The reaction mixture was heated at reflux for 16 h. The solvent was removed by rotary evaporation. Purification by flash column chromatography (SiO_2, *n*-pentane : EtOAc, 10 : 1) yielded **15** as a yellow solid (3.6 g, 2.3 mmol, quant.). ^{1}H NMR (400 MHz, $CDCl_3$): δ = 7.81 – 7.63 (m, 10H), 7.44 – 7.32 (m, 10H), 6.81 (t, J = 7.9 Hz, 4H), 6.67 (d, J = 5.1 Hz, 2H), 6.65 (d, J = 5.2 Hz, 2H), 6.44 (d, J = 7.8 Hz, 2H), 6.39 (d, J = 7.8 Hz, 2H), 4.36 – 4.25 (m, 2H), 4.15 (dt, J = 16.1, 5.6 Hz, 4H), 4.06 – 3.90 (m, 6H), 3.82 (m, 8H), 3.75 – 3.58 (m, 8H), 1.91 (m, 4H), 1.59 (m, 4H), 1.35 (m, 32H), 1.06 (s, 18H), 0.97 – 0.76 (m, 6H) ppm; ^{13}C NMR (100.6 MHz, $CDCl_3$): δ = 155.4, 155.1, 137.0, 136.6, 135.8, 133.9, 129.8, 127.9, 126.0, 125.9, 125.0, 122.8, 122.3, 109.6, 109.3, 72.7, 71.4, 71.1, 69.9, 69.2, 68.8, 63.6, 32.2, 30.0, 29.9, 29.8, 29.6, 29.5, 27.1, 26.4, 22.9, 19.4, 14.4 ppm; HRMS-ESI- *m/z* calculated for $C_{94}H_{123}O_{10}S_2Si_2Cl$ $[M - H + Cl]^-$: 1567.7813, found 1567.7879.

2,2'-((((((4',5'-Bis(dodecyloxy)-[9,9'-bithioxanthenylidene]-4,5-diyl)bis(oxy))bis(ethane-2,1-diyl))bis(oxy))bis(ethane-2,1-diyl))bis(oxy))diethanol (1):

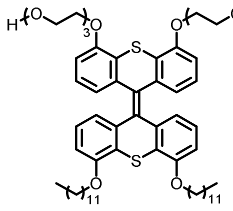

Compound **15** (90 mg, 0.059 mmol, 1.0 equiv) was dissolved in THF (5 mL) and TBAF (1 M in THF) (0.15 mL, 0.15 mmol, 2.5 equiv) was added at 0 °C. The reaction mixture was stirred at RT for 16 h. The solvent was removed by rotary evaporation and the residue purified by flash column chromatography (SiO_2, 5% MeOH in EtOAc) to yield **1** as an off-white solid (56 mg, 0.053 mmol, 90%). ^1H NMR (400 MHz, $CDCl_3$): δ = 6.82 (dd, *J* = 14.5, 7.8 Hz, 4H), 6.66 (t, *J* = 7.3 Hz, 4H), 6.44 (d, *J* = 7.7 Hz, 2H), 6.38 (d, *J* = 7.7 Hz, 2H), 4.31 (d, *J* = 4.4 Hz, 2H), 4.20 (d, *J* = 4.2 Hz, 2H), 4.13 (d, *J* = 9.0 Hz, 2H), 4.00 (dd, *J* = 9.5, 6.2 Hz, 6H), 3.90 (t, *J* = 4.6 Hz, 4H), 3.76 (d, *J* = 3.1 Hz, 8H), 3.70 – 3.63 (m, 4H), 2.95 (s, 1H), 1.96 – 1.85 (m, 4H), 1.70 (d, *J* = 2.8 Hz, 1H), 1.57 (dd, *J* = 13.9, 6.6 Hz, 4H), 1.43 – 1.21 (m, 32H), 0.88 (t, *J* = 6.7 Hz, 6H) ppm; ^{13}C NMR (75.4 MHz, $CDCl_3$): δ = 155.4, 155.0, 137.2, 136.5, 133.5, 132.8, 126.1, 125.9, 125.0, 124.8, 122.9, 122.3, 118.7, 109.5, 109.3, 73.0, 71.4, 70.8, 69.9, 69.2, 69.0, 62.0, 32.2, 29.9, 29.7, 29.63, 29.5, 26.4, 22.9, 14.4 ppm; HRMS-APCI+ *m*/*z* calculated for $C_{62}H_{89}O_{10}S_2$ $[M + H]^+$ 1057.5897, found 1057.5889.

2.2 Synthesis of chiral 2

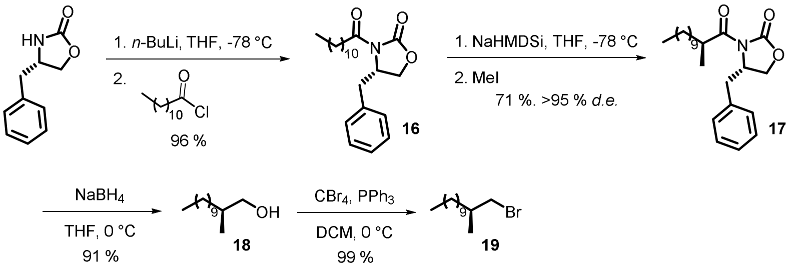

Scheme S5. Synthesis of chiral alkylhalide **19**.

(*S*)-4-Benzyl-3-dodecanoyloxazolidin-2-one (16):

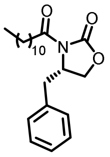

This compound was prepared as described in literature.[3] A solution of (*S*)-4-benzyloxazolidin-2-one (3.20 g, 18.1 mmol, 1.0 equiv) in THF (100 mL) at -78 °C was treated dropwise with *n*-BuLi (12.4 ml, 19.9 mmol, 1.1 equiv) to give a colored light yellow solution. After 15 min, dodecanoyl chloride (4.54 g, 20.8 mmol, 1.2 equiv) was added. The resulting mixture was left to warm to room temperature over 1 h. The reaction was quenched by addition of 1 M aq. NH_4Cl (100 mL). Diethylether (100 mL) was added and the layers separated. The water phase was washed with diethylether (100 mL) and the combined organic phase was dried over Na_2SO_4. The solvent was evaporated under reduced pressure and the residue purified by flash column chromatography (SiO_2, *n*-pentane : EtOAc, 15 : 1) to give **16** as a white solid (6.24 g, 17.4 mmol, 96%). m.p. 46 – 47 °C; ^{1}H NMR (300 MHz, $CDCl_3$): δ = 7.26 – 7.33 (m, 3H), 7.21 (d, *J* = 7.2 Hz, 2H), 7.13 (d, *J* = 7.9 Hz, 2H), 4.68 (m, 1H), 4.18 (m, 2H), 3.30 (dd, *J* = 13.3, 3.1 Hz, 1H), 2.93 (m, 2H), 2.77 (dd, *J* = 13.3, 9.6 Hz, 1H), 1.69 (m, 2H), 1.27 (m, 16H), 0.88 (t, *J* = 6.8 Hz, 3H) ppm; ^{13}C NMR (75.4 MHz, $CDCl_3$): δ = 173.6, 153.7, 135.5, 129.6, 129.1, 127.5, 66.3, 55.4, 38.1, 35.8, 32.1, 29.8, 29.7, 29.6, 29.5, 29.3, 24.5, 22.9, 14.3 ppm. Data is in accordance with literature.[3]

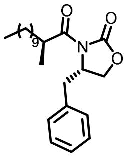

(*S*)-4-Benzyl-3-((*S*)-2-methyldodecanoyl)oxazolidin-2-one (17): This compound was prepared as described in literature.[4] A solution of **16** (6.20 g, 17.3 mmol, 1.0 equiv) in THF (150 ml) at -78 °C was treated with NaHMDS (22.4 mL, 22.4 mmol, 1.3 equiv, 1 M in THF) to give a light yellow colored solution. After 30 min, MeI (5.40 mL, 86.0 mmol, 5.0 equiv) was added and the resulting mixture was stirred at -78 °C for 30 min and then left to warm to room temperature over 1.5 h. The reaction was quenched with 1 M aq. NH_4Cl (100 mL) and the mixture was extracted with diethylether (2 × 100 mL). The combined organic phase was dried with Na_2SO_4 and the solvents evaporated under reduced pressure. The residue was purified by flash column chromatography (SiO_2, *n*-pentane : EtOAc, 30 : 1 to 10 : 1) to yield **17** as a colorless oil (4.54 g, 12.3 mmol, 71%, > 95% *d.e.* as determined by ^{1}H NMR). ^{1}H NMR (300 MHz, $CDCl_3$): δ = 7.26 – 7.33 (m, 3H), 7.21 (d, *J* = 7.2 Hz, 2H), 7.13 (d, *J* = 7.9 Hz, 2H), 4.67 (m, 1H), 4.19 (m, 2H), 3.70 (m, 1H), 3.27 (dd, J_1 = 13.3, 3.2 Hz, 1H), 2.93 (m, 1H), 2.77 (dd, *J* = 13.3, 9.6 Hz, 1H), 1.73 (m, 1H), 1.42 (m, 1H), 1.27 (m, 19H), 0.88 (t, *J* = 6.8 Hz, 3H) ppm; ^{13}C NMR (75.4 MHz, $CDCl_3$): δ = 177.6, 153.3, 135.5, 129.6, 129.1, 127.5, 66.2, 55.6, 38.1, 37.9, 33.6, 32.1, 29.9, 29.8, 29.7, 29.5, 27.5, 22.9, 17.6, 14.3 ppm. Data is in accordance with literature.[4]

(*S*)-2-Methyldodecan-1-ol (18):

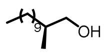

This compound was prepared as described in literature.[5] To a solution of **17** (4.20 g, 11.2 mmol, 1.0 equiv) in THF (150 mL) at 0 °C, $NaBH_4$ (735 mg, 33.7 mmol, 3.0 equiv) was

added in portions. After stirring for 2 h, the reaction was quenched with 1 M aq. Rochelle's salt (100 mL) and extracted with diethylether (2 × 100 mL). The combined organic phase was dried over Na_2SO_4 and the solvents evaporated under reduced pressure. The residue was purified by flash column chromatography (SiO_2, *n*-pentane : EtOAc, 10 : 1) to yield **18** as a colorless oil (2.06 g, 10.2 mmol, 91%). $[\alpha]_D$ = –7.8 (c 0.051, $CHCl_3$); ^{1}H NMR (300 MHz, $CDCl_3$): δ = 3.52 (dd, *J* = 10.5, 5.8 Hz, 1H), 3.42 (dd, *J* = 10.5, 6.6 Hz, 1H), 1.61 (m, 1H), 1.27 (m, 18H), 1.11 (m, 1H), 0.88 (t, *J* = 6.8 Hz, 3H) ppm; ^{13}C NMR (75.4 MHz, $CDCl_3$): δ = 68.7, 36.0, 33.4, 32.1, 30.2, 29.9, 29.87, 29.86, 29.6, 27.2, 22.9, 16.8, 14.4 ppm. Data is in accordance with literature.[5]

(*S*)-1-Bromo-2-methyldodecane (19):

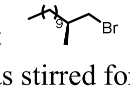

This compound was prepared as described in literature.[6] To a solution of **18** (2.00 g, 10.0 mmol, 1.0 equiv) and CBr_4 (3.97 g, 12.0 mmol, 1.2 equiv) in CH_2Cl_2 (150 mL) at 0 °C, PPh_3 (3.14 g, 12.0 mmol, 1.2 equiv) was added in portions. The resulting solution was stirred for 2 h. Water (80 mL) was added and the layers were separated. The water phase was extracted with CH_2Cl_2 (80 mL). The combined organic extracts were dried over Na_2SO_4, the solvents evaporated under reduced pressure and the residue purified by flash column chromatography (SiO_2, *n*-hexane) to yield **19** as a colorless oil (2.61 g, 9.90 mmol, 99%), $[\alpha]_D$ = –0.27 (c 0.407, $CHCl_3$); ^{1}H NMR (300 MHz, $CDCl_3$): δ = 3.41 (dd, *J* = 9.8, 4.9 Hz, 1H), 3.42 (dd, *J* = 9.8, 6.2 Hz, 1H), 1.78 (m, 1H), 1.44 (m, 1H), 1.27 (m, 16H), 1.02 (d, *J* = 6.6 Hz, 3H), 0.88 (t, *J* = 6.8 Hz, 6H) ppm; ^{13}C NMR (75.4 MHz, $CDCl_3$): δ = 41.9, 35.4, 35.1, 32.1, 29.9, 29.85, 29.81, 29.6, 27.1, 22.9, 19.0, 14.3 ppm. Data is in accordance with literature.[6]

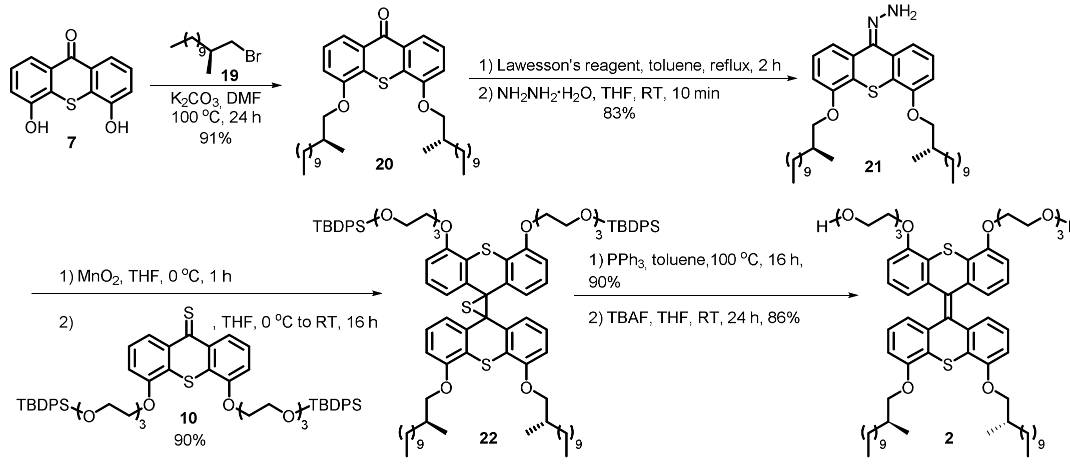

Scheme S6. Synthesis of chiral amphiphile **2**.

4,5-Bis(((*S*)-2-methyldodecyl)oxy)-9*H*-thioxanthen-9-one (20):

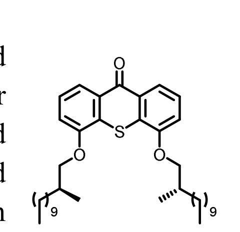

A mixture of **7** (0.93 g, 3.8 mmol, 1.0 equiv), **19** (2.6 g, 9.9 mmol, 2.6 equiv) and K_2CO_3 (2.6 g, 19 mmol, 5.0 equiv) in DMF (20 mL) was heated to 100 °C for 24 h. The DMF was evaporated under reduced pressure and water (80 mL) and CH_2Cl_2 (150 mL) were added to the residue. The organic phase was separated and concentrated under reduced pressure. The residue was purified by flash column chromatography (SiO_2, *n*-pentane : EtOAc, 10 : 1) to yield **20** as a yellow solid (2.1 g, 3.5 mmol, 91%). m.p. 63 – 64 °C; ^{1}H NMR (300 MHz, $CDCl_3$): δ = 7.68 (dd, *J* = 7.6, 1.7 Hz, 8H), 7.32 – 7.40 (m, 12H), 7.30 (d, *J* = 7.8 Hz, 2H), 7.24 (d, *J* = 8.1 Hz, 2H), 6.83 (dd, *J* = 8.0, 8.0 Hz, 2H), 6.82 (dd, *J* = 8.0, 8.0 Hz, 2H), 6.55 (d, *J* = 8.1 Hz, 2H), 6.48 (d, *J* = 8.1 Hz, 2H), 4.04 (m 2H), 3.96 (m, 2H), 3.48 – 3.86 (m, 24H), 1.90 (m, 2H), 1.43 – 1.52 (m, 2H), 1.27 (m, 32H), 1.05 (m,

21H), 1.00 (d, J = 6.7 Hz, 3H), 0.88 (t, J = 6.9 Hz, 6H) ppm; ^{13}C NMR (75.4 MHz, $CDCl_3$): δ = 180.4, 154.5, 130.0, 128.6, 125.8, 121.2, 112.9, 74.3, 33.5, 33.3, 31.9, 30.0, 29.7, 29.68, 29.4, 27.0, 22.7, 17.1, 14.1 ppm; HRMS-APCI+ m/z calculated for $C_{39}H_{61}O_3S$ $[M + H]^+$ 609.4341, found 609.4336.

(4,5-Bis(((*S*)-2-methyldodecyl)oxy)-9*H*-thioxanthen-9-ylidene)hydrazine (21):

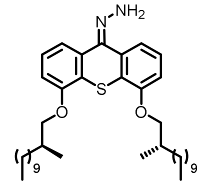

A mixture of **20** (800 mg, 1.31 mmol, 1.0 equiv) and Lawesson's reagent (797 mg, 1.97 mmol, 1.5 equiv) in toluene (40 mL) was refluxed under nitrogen atmosphere for 2 h. The resulting yellow solution was cooled to room temperature and filtered through a plug of silica gel. The plug was washed with CH_2Cl_2 until the filtrate was colorless. The solvents were evaporated and the residue was redissolved in THF (30 mL). The solution was treated with aq. 40% hydrazine (1.60 ml, 25.5 mmol, 19 equiv) at room temperature. The solution decolorized within several min. The solvent and excess hydrazine were removed under reduced pressure. The residue was purified by flash column chromatography (SiO_2, n-pentane : EtOAc, 10 : 1) to yield **21** as a yellow oil (681 mg, 1.09 mmol, 83%). ^{1}H NMR (300 MHz, $CDCl_3$): δ = 7.64 (dd, J = 7.9, 0.9 Hz, 1H), 7.44 (dd, J = 7.9, 0.9 Hz, 1H), 7.28 (dd, J = 8.0, 8.0 Hz, 1H), 7.24 (dd, J = 8.0, 8.0 Hz, 1H), 6.88 (dd, J = 8.1, 0.8 Hz, 1H), 6.82 (dd, J = 8.1, 1.0 Hz, 1H), 5.85 (br s, 2H), 3.96 (m, 2H), 3.85 (m, 2H), 2.06 (m, 2H), 1.62 (m, 2H), 1.27 (m, 32H), 1.14 (d, J = 6.7 Hz, 3H), 1.12 (d, J = 6.7 Hz, 3H), 0.89 (t, J = 6.8 Hz, 6H) ppm; ^{13}C NMR (75.4 MHz, $CDCl_3$): δ = 155.9, 154.4, 142.0, 135.2, 126.9, 126.6, 125.6, 125.6, 121.9, 120.0, 118.3, 111.0, 110.0, 74.2, 74.2, 33.7, 33.68, 33.5, 32.1, 30.2, 30.17, 29.9, 29.89, 29.6, 27.3, 27.26, 22.9, 17.4, 14.3 ppm; HRMS-APCI+ m/z calculated for $C_{39}H_{63}N_2O_2S$ $[M + H]^+$ 623.4610, found 623.4605.

Episulfide 22:

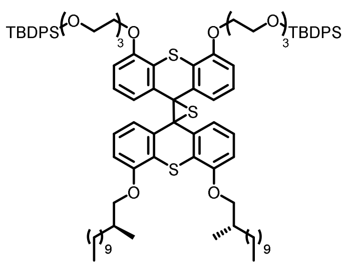

Solid MnO_2 (347 mg, 4.00 mmol, 10 equiv) was added to a solution of **21** (299 mg, 0.480 mmol, 1.2 equiv) in THF (50 mL) at 0 °C. The resulting mixture was stirred for 1 h at 0 °C and filtered through a plug of silica gel. The plug was washed with a small amount of THF (10 mL). The light green solution was cooled back to 0 °C. A solution of **10** (400 mg, 0.400 mmol, 1.0 equiv) in THF (2.4 mL) was added dropwise and the resulting mixture was stirred overnight. The solvents were evaporated under reduced pressure and the residue purified by flash column chromatography (SiO_2, n-pentane : EtOAc, 5 : 1) to yield **22** as a light yellow oil (570 mg, 0.360 mmol, 90%). ^{1}H NMR (300 MHz, $CDCl_3$): δ = 7.68 (dd, J = 7.6, 1.7 Hz, 8H), 7.32 – 7.40 (m, 12H), 7.30 (d, J = 7.8 Hz, 2H), 7.24 (d, J = 8.1 Hz, 2H), 6.83 (dd, J = 8.0, 8.0 Hz, 2H), 6.82 (dd, J = 8.0, 8.0 Hz, 2H), 6.55 (d, J = 8.1 Hz, 2H), 6.48 (d, J = 8.1 Hz, 2H), 4.04 (m 2H), 3.96 (m, 2H), 3.48 – 3.86 (m, 24H), 1.90 (m, 2H), 1.43 – 1.52 (m, 2H), 1.27 (m, 32H), 1.05 (m, 21H), 1.00 (d, J = 6.7 Hz, 3H), 0.88 (t, J = 6.9 Hz, 6H) ppm; ^{13}C NMR (75.4 MHz, $CDCl_3$): δ = 154.2, 154.1, 153.81, 135.78, 133.9, 132.62, 132.60, 132.04, 132.00, 129.8, 127.8, 126.1, 126.0, 125.7, 125.1, 124.9, 124.01, 124.00, 123.2, 123.1, 113.9, 111.7, 111.6, 110.1, 110.0, 74.6, 74.5, 72.6, 71.2, 71.0, 69.7, 69.4, 69.38, 66.7, 63.6, 33.7, 33.6, 33.4, 33.36, 32.1, 30.2, 30.0, 29.95, 29.93, 29.90, 29.88, 29.6, 27.3, 27.1, 27.0, 22.9, 19.4, 17.5, 17.1, 14.3 ppm; HRMS-APCI+ m/z calculated for $C_{96}H_{128}O_{10}S_3Si_2Na$ $[M + Na]^+$ 1616.8139, found 1616.8134.

12,12'-((4',5'-Bis(((*S*)-2-methyldodecyl)oxy)-[9,9'-bithioxanthenylidene]-4,5-diyl)bis(oxy))bis(2,2-dimethyl-3,3-diphenyl-4,7,10-trioxa-3-siladodecane) (23):

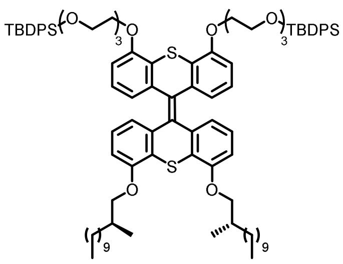

PPh_3 (407 mg, 1.55 mmol, 5.0 equiv) was added to a solution of **22** (495 mg, 0.310 mmol, 1.0 equiv) in toluene (40 mL). The resulting mixture was heated to 100 °C overnight. The solvents were evaporated under reduced pressure and the residue purified by flash column chromatography (SiO_2, *n*-pentane:EtOAc, 5:1) to yield **23** as a colorless oil (437 mg, 0.279 mmol, 90%). ^{1}H NMR (300 MHz, $CDCl_3$): δ = 7.68 (dd, *J* = 7.6, 1.9 Hz, 8H), 7.31 – 7.39 (m, 12H), 6.78 (m, 4H), 6.62 (m, 4H), 6.42 (d, *J* = 7.7 Hz, 2H), 6.37 (dd, *J* = 7.8, 0.8 Hz, 2H), 4.26 (m 2H), 4.14 (m, 2H), 3.62 – 3.93 (m, 24H), 2.05 (m, 2H), 1.59 – 1.66 (m, 2H), 1.27 (m, 32H), 1.14 (m, 6H), 1.04 (s, 18H), 0.88 (t, *J* = 6.9 Hz, 6H) ppm; ^{13}C NMR (75.4 MHz, $CDCl_3$): δ = 155.45, 155.43, 155.1, 137.04, 137.03, 136.49, 136.48, 135.8, 133.9, 133.3, 132.8, 129.8, 127.8, 125.9, 125.8, 125.2, 125.1, 124.9, 122.7, 122.3, 122.2, 109.6, 109.2, 109.1, 74.2, 74.0, 72.7, 71.3, 71.1, 69.9, 68.8, 63.6, 33.8, 33.6, 33.59, 33.5, 32.1, 30.2, 30.1, 30.0, 29.95, 29.91, 29.88, 29.87, 29.6, 27.3, 27.28, 27.0, 22.9, 19.4, 17.6, 17.3, 14.3 ppm; HRMS-APCI+ *m*/*z* calculated for $C_{96}H_{128}O_{10}S_2Si_2Na$ $[M + Na]^+$ 1584.8419, found 1584.8413.

2,2'-((((((4',5'-Bis(((*S*)-2-methyldodecyl)oxy)-[9,9'-bithioxanthenylidene]-4,5-diyl)bis(oxy))bis(ethane-2,1-diyl))bis(oxy))bis(ethane-2,1-diyl))bis(oxy))diethanol (2):

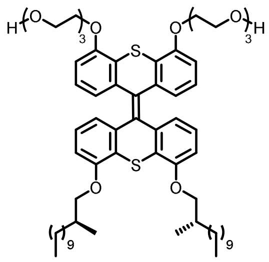

A solution of **23** (75 mg, 0.050 mmol, 1.0 equiv) in THF (5 mL) was treated dropwise with TBAF (143 µL, 0.140 mmol, 2.8 equiv, 1 M in THF) at room temperature. After 24 h, the solvents were evaporated under reduced pressure and the residue purified by flash column chromatography (SiO_2, EtOAc:MeOH, 10:1) and subsequently recrystallized from methanol (3 mL) to yield **2** as a white solid (45 mg, 0.040 mmol, 86%). mp 150 – 152 °C; ^{1}H NMR (300 MHz, $CDCl_3$): δ = 6.84 (dd, *J* = 8.0, 8.0 Hz, 2H), 6.82 (dd, *J* = 8.0, 8.0 Hz, 2H), 6.68 (d, *J* = 8.6 Hz, 2H), 6.65 (d, *J* = 8.3 Hz, 2H), 6.45 (d, *J* = 7.8 Hz, 2H), 6.38 (d, *J* = 7.6 Hz, 2H), 4.31 (m 2H), 4.22 (m, 2H), 3.67 – 4.02 (m, 24H), 2.06 (m, 2H), 1.58 – 1.68 (m, 2H), 1.28 (m, 32H), 1.15 (m, 6H), 1.04 (s, 18H), 0.89 (t, *J* = 6.6 Hz, 6H) ppm; ^{13}C NMR (75.4 MHz, $CDCl_3$): δ = 155.50, 155.48, 155.00, 137.32, 137.31, 136.393, 136.386, 133.5, 132.7, 126.1, 125.8, 125.2, 125.1, 124.7, 122.9, 122.23, 122.21, 109.5, 109.3, 109.2, 74.2, 74.04, 73.0, 71.4, 70.7, 69.9, 68.89, 61.9, 33.8, 33.7, 33.6, 33.5, 32.1, 30.2, 30.17, 29.99, 29.97, 29.94, 29.90, 29.89, 29.6, 27.4, 27.3, 22.9, 17.6, 17.4, 14.3 ppm; HRMS-APCI+ *m*/*z* calculated for $C_{64}H_{92}O_{10}S_2Na$ $[M + Na]^+$ 1107.6030, found 1107.6024.

2.3 NMR spectra of all new compounds

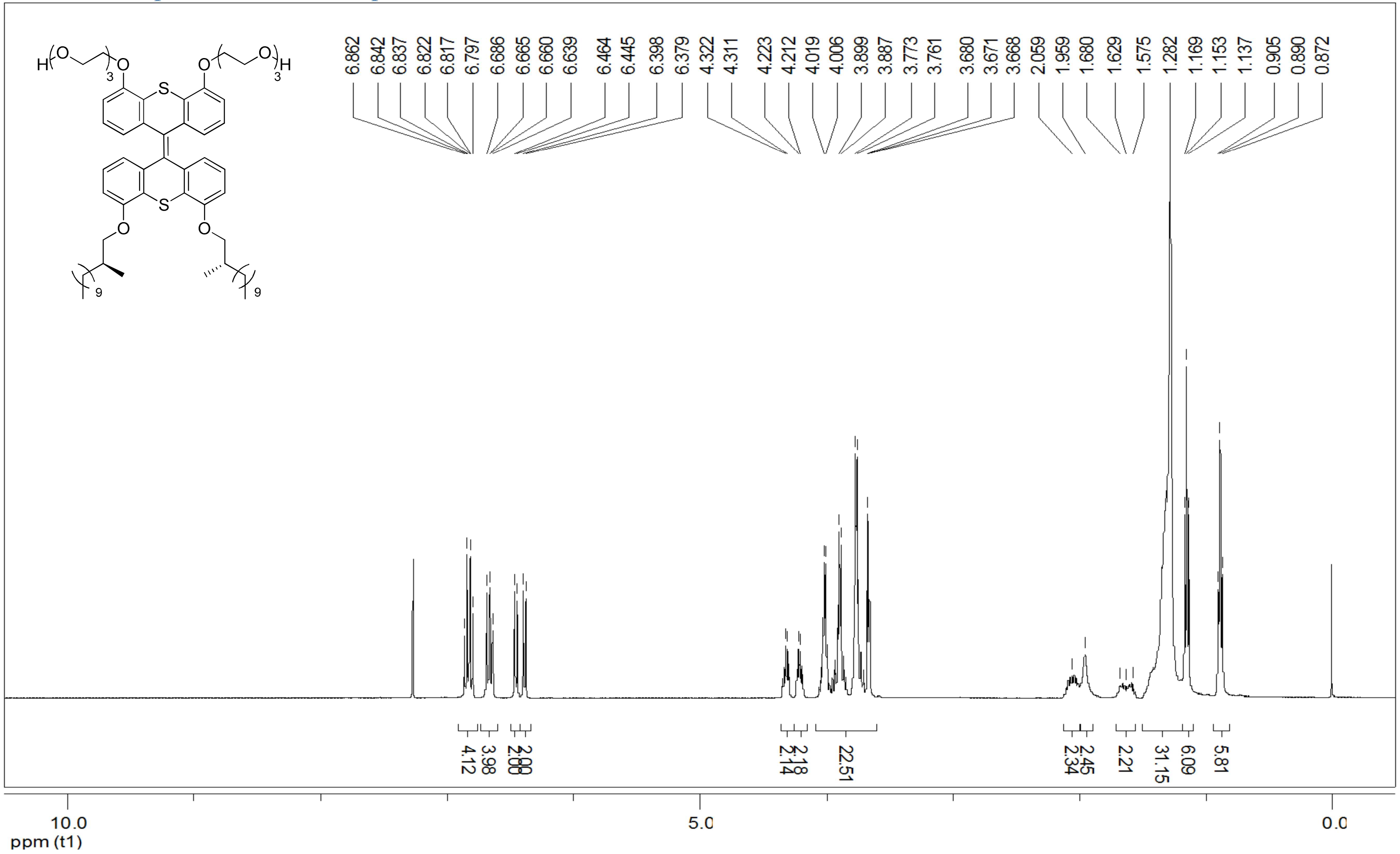

Figure S1. ^{1}H NMR spectrum of **2** in $CDCl_3$ at 400 MHz.

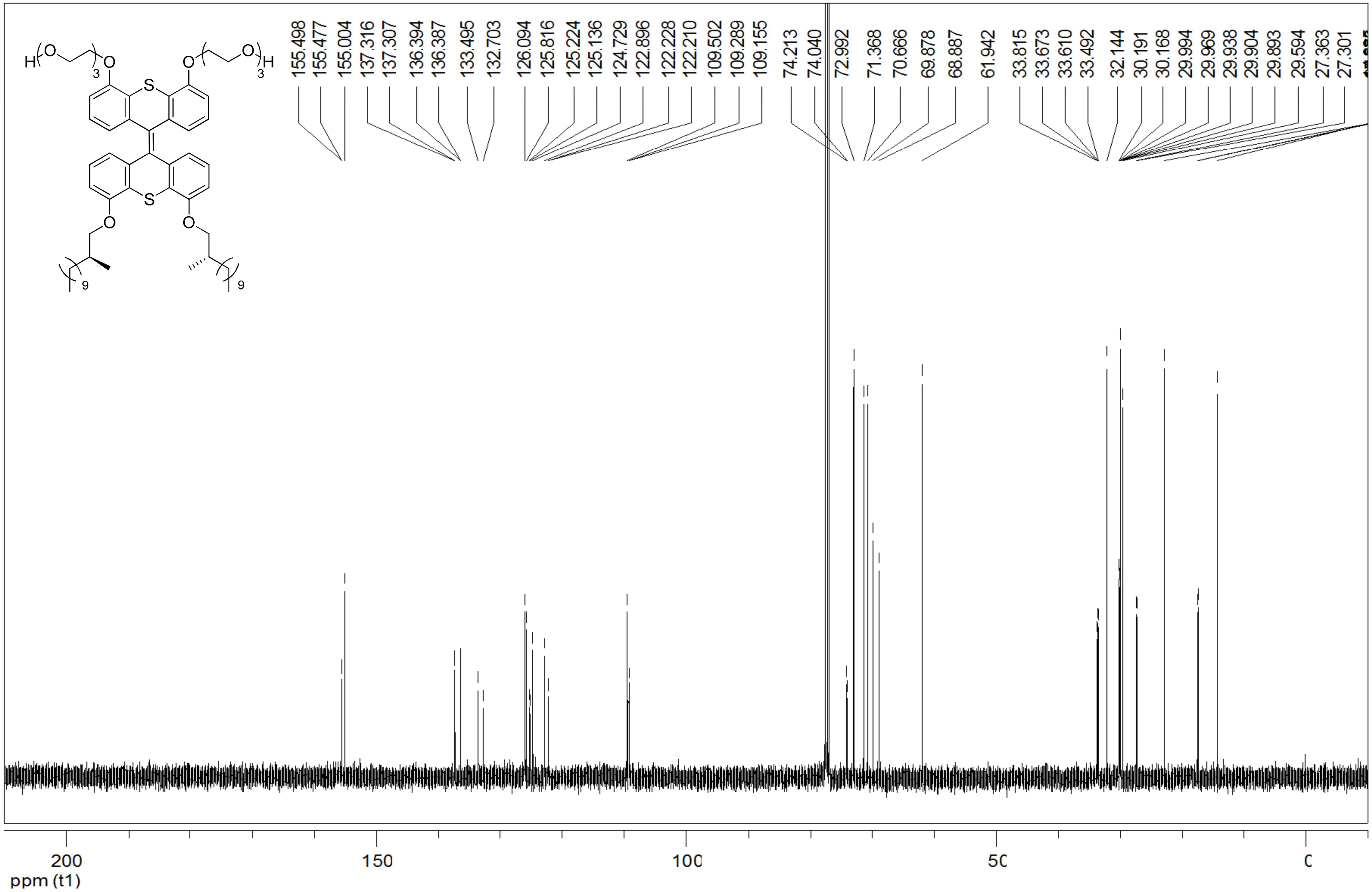

Figure S2. ^{13}C NMR spectrum of **2** in $CDCl_3$ at 100.6 MHz.

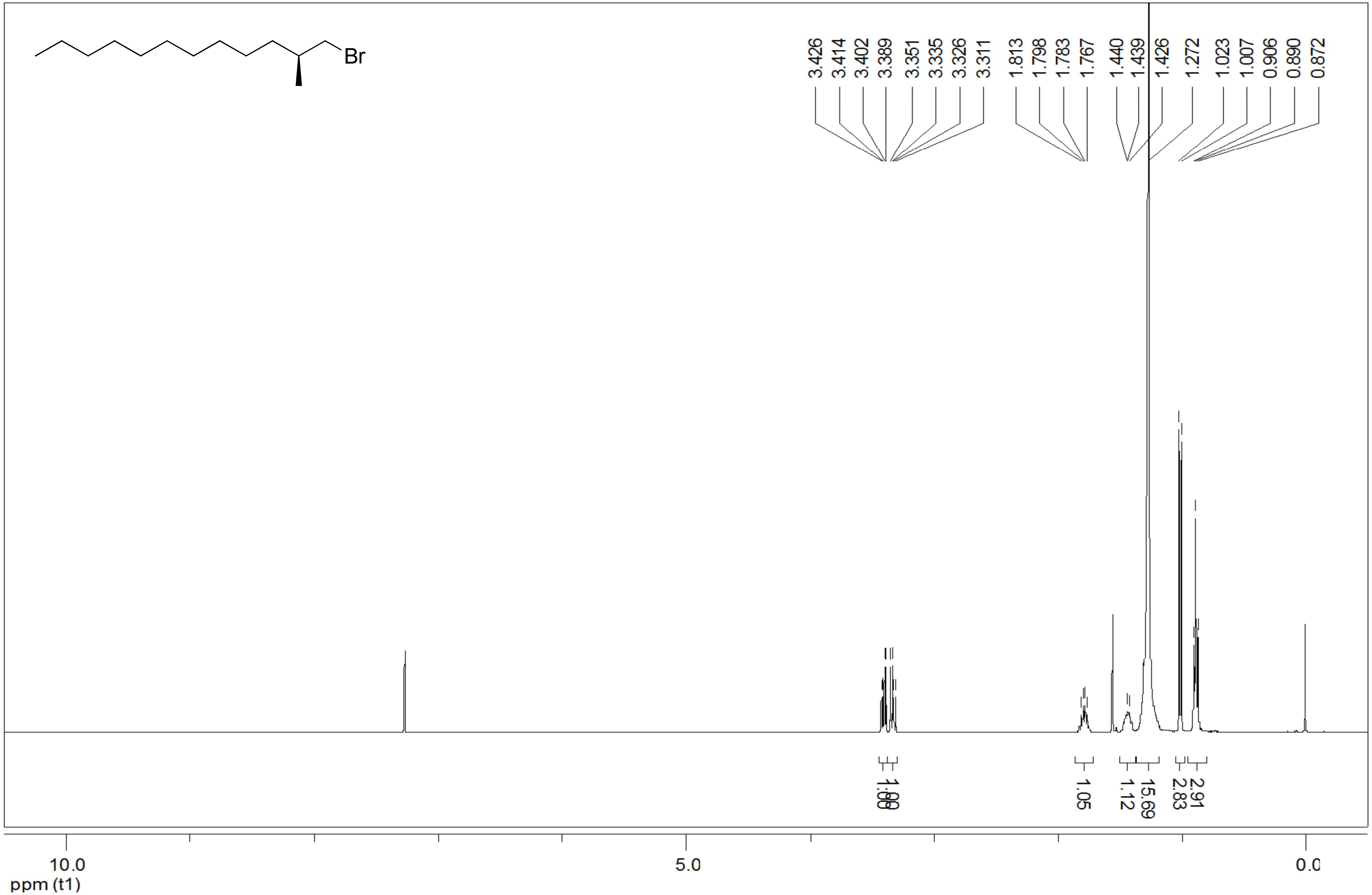

Figure S3. ^{1}H NMR spectrum of **19** in $CDCl_3$ at 400 MHz.

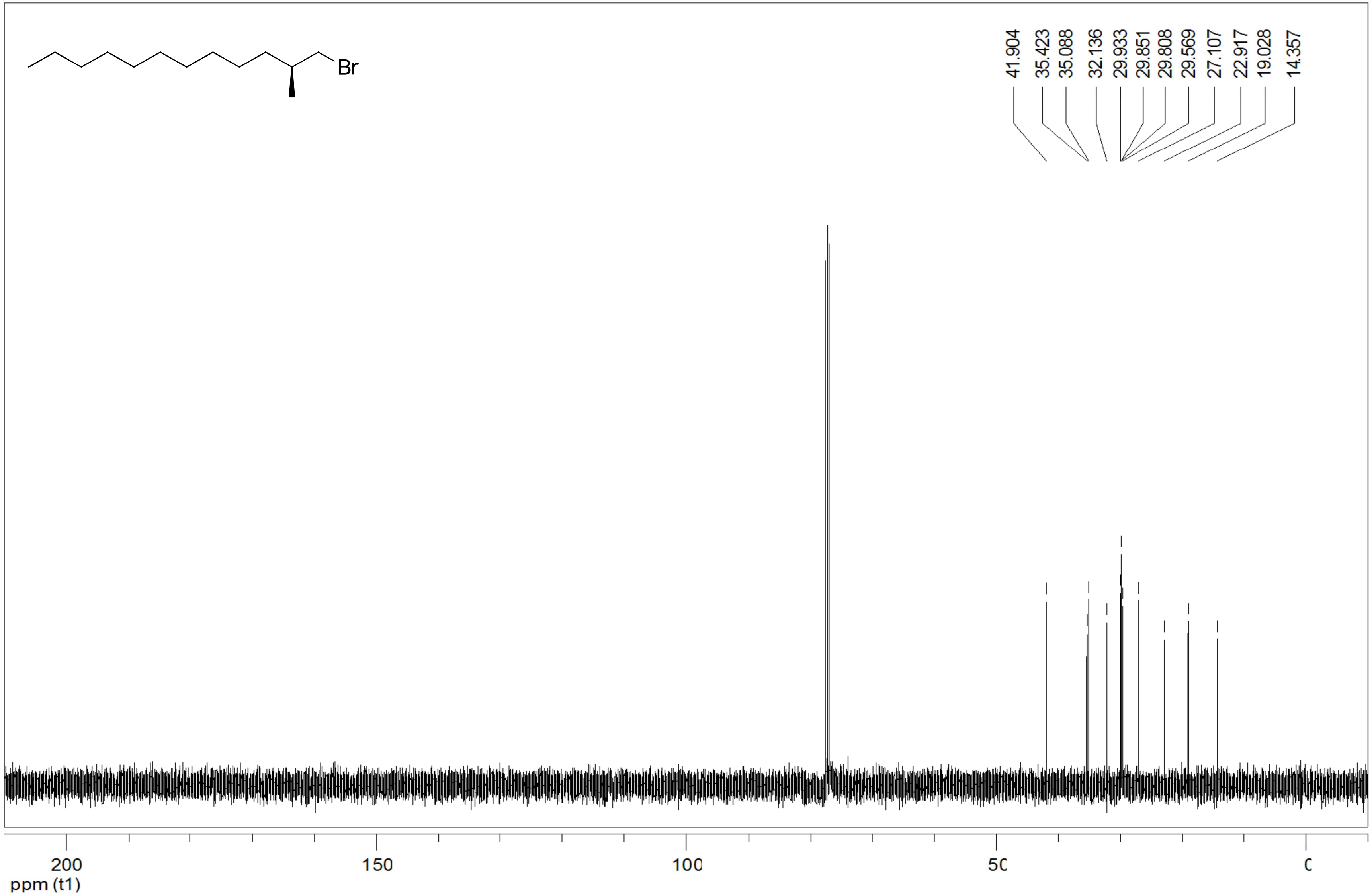

Figure S4. ^{13}C NMR spectrum of **19** in $CDCl_3$ at 400 MHz.

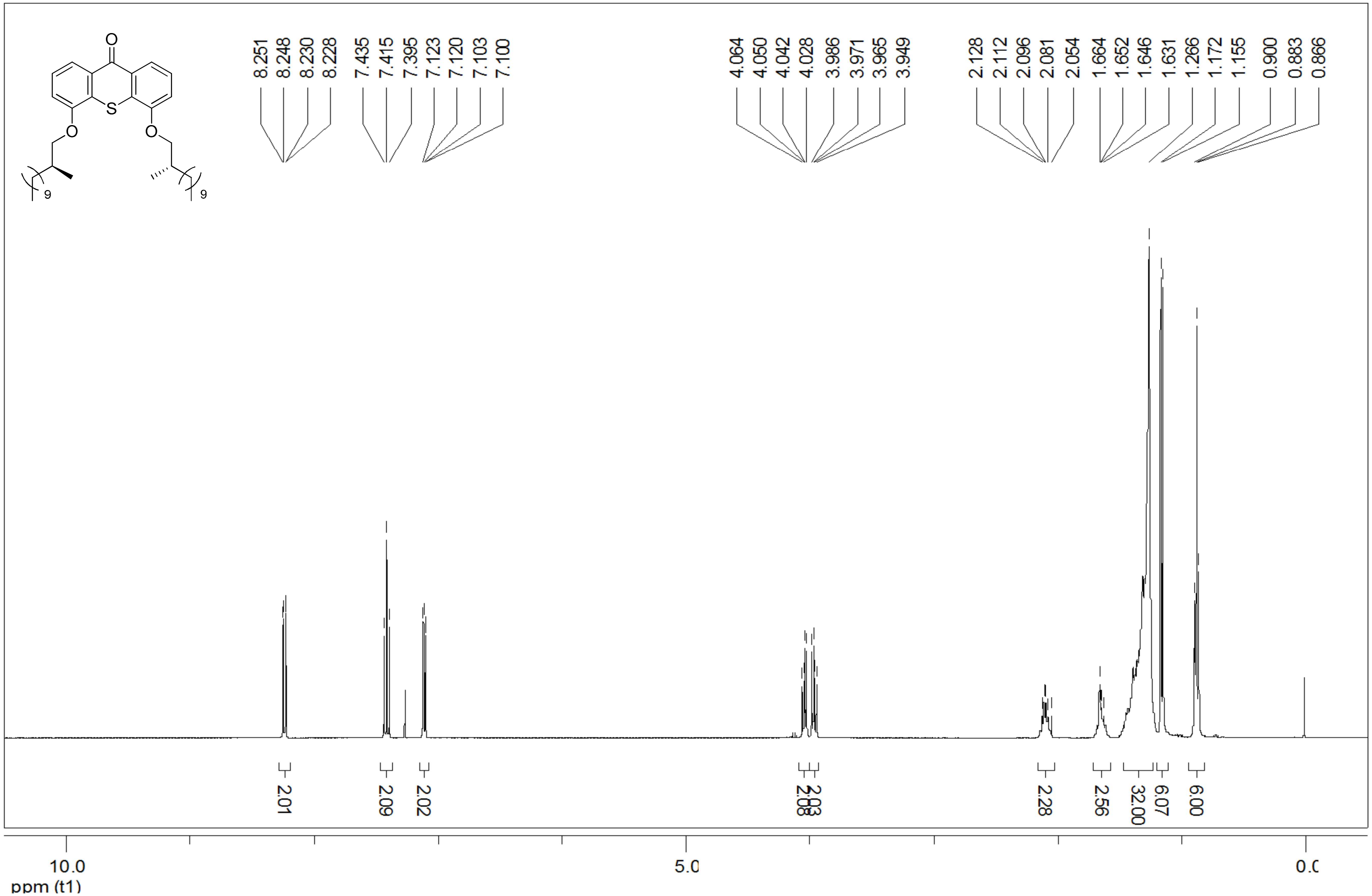

Figure S5. ^{1}H NMR spectrum of **20** in $CDCl_3$ at 400 MHz.

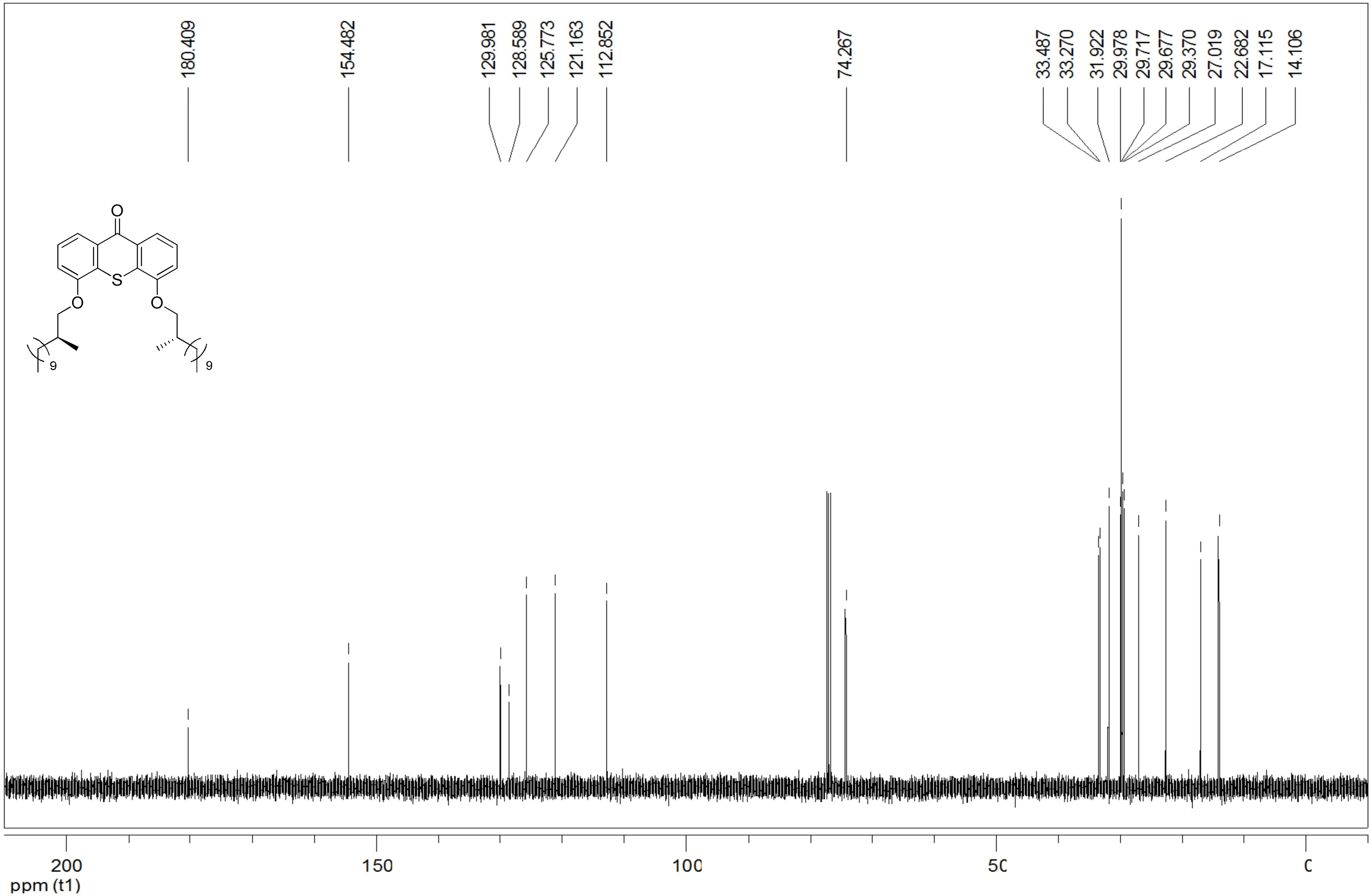

Figure S6. ^{13}C NMR spectrum of **20** in $CDCl_3$ at 100.6 MHz.

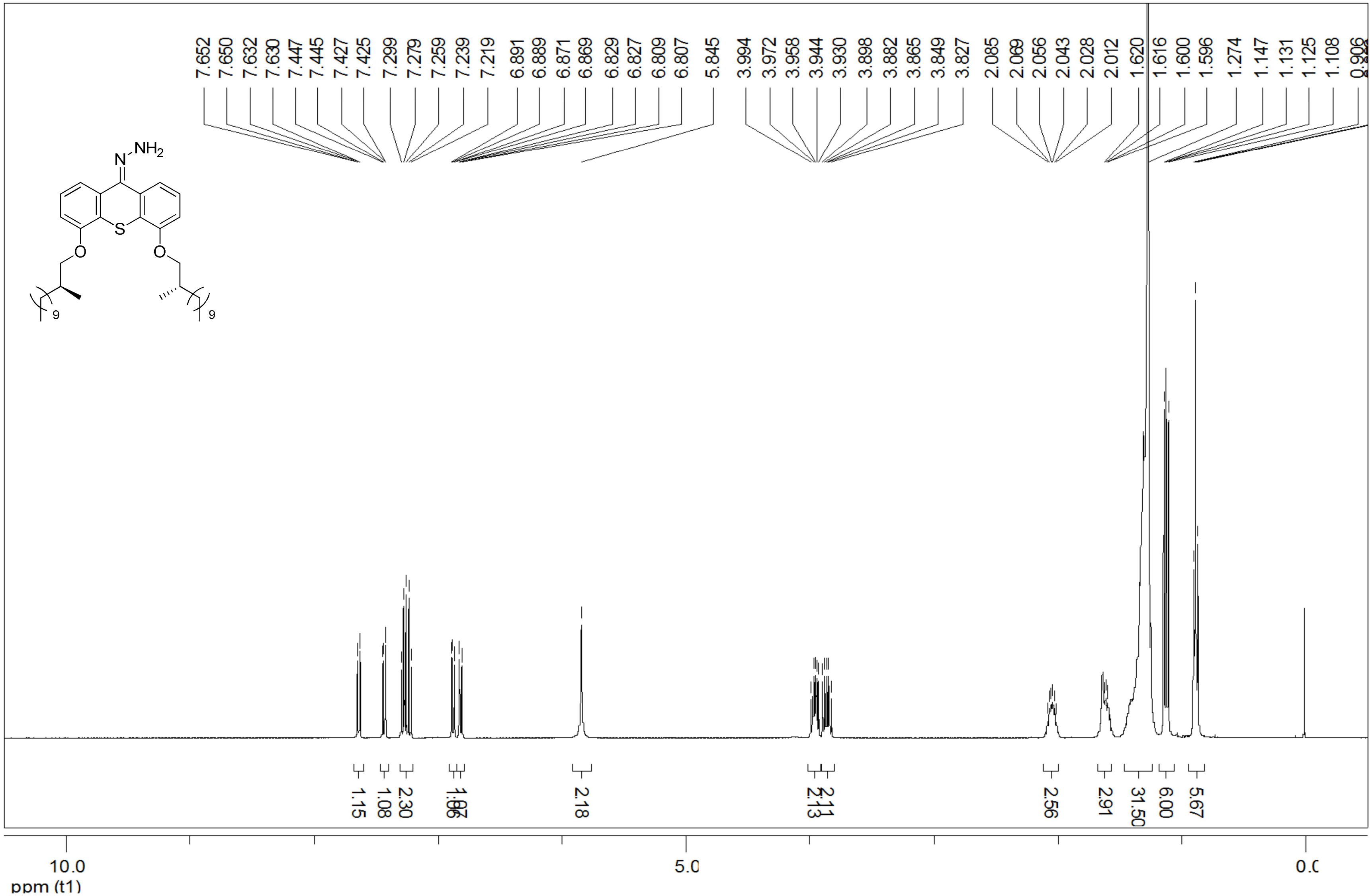

Figure S7. ^{1}H NMR spectrum of **21** in $CDCl_3$ at 400 MHz.

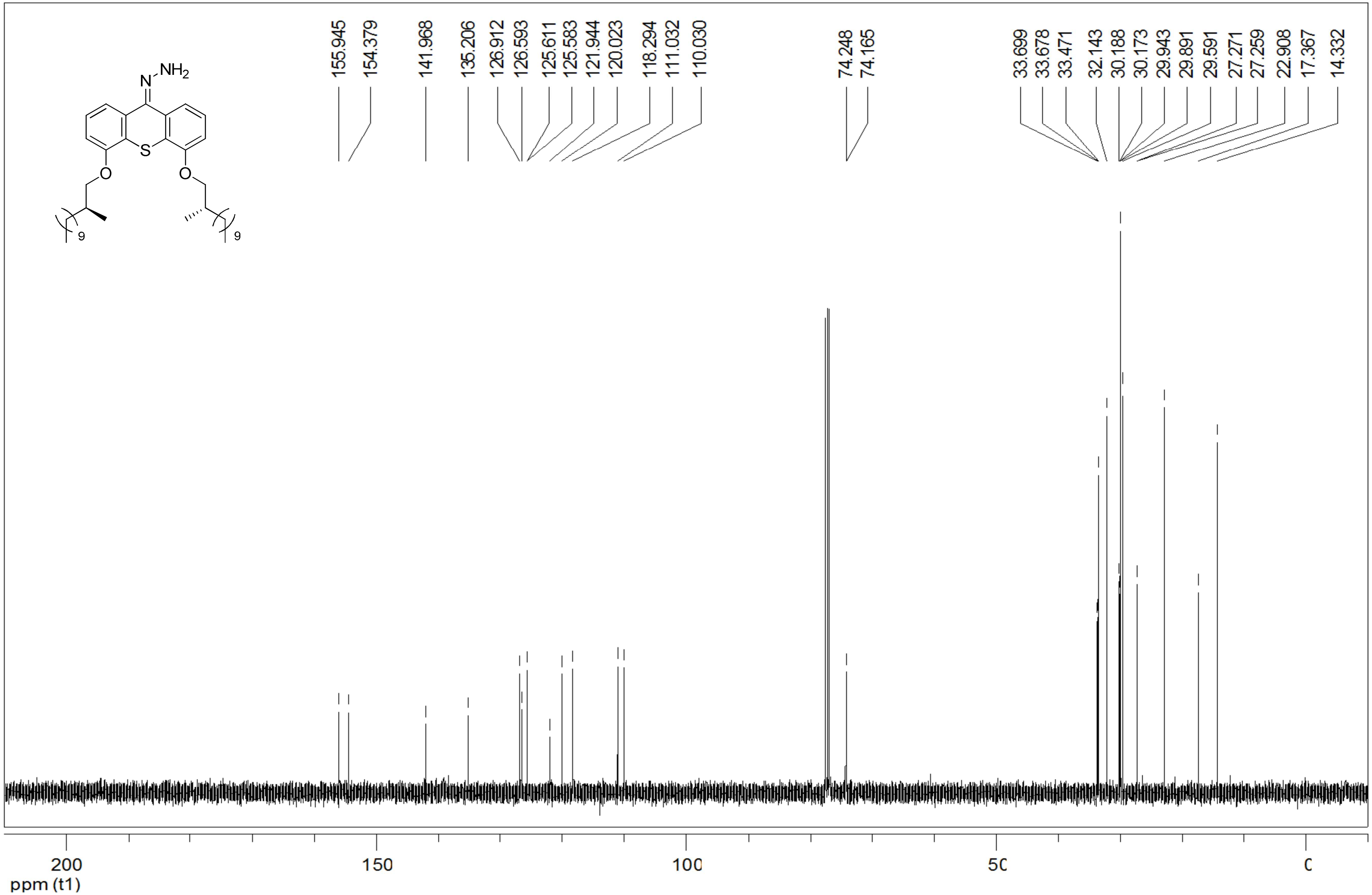

Figure S8. ^{13}C NMR spectrum of **21** in $CDCl_3$ at 100.6 MHz.

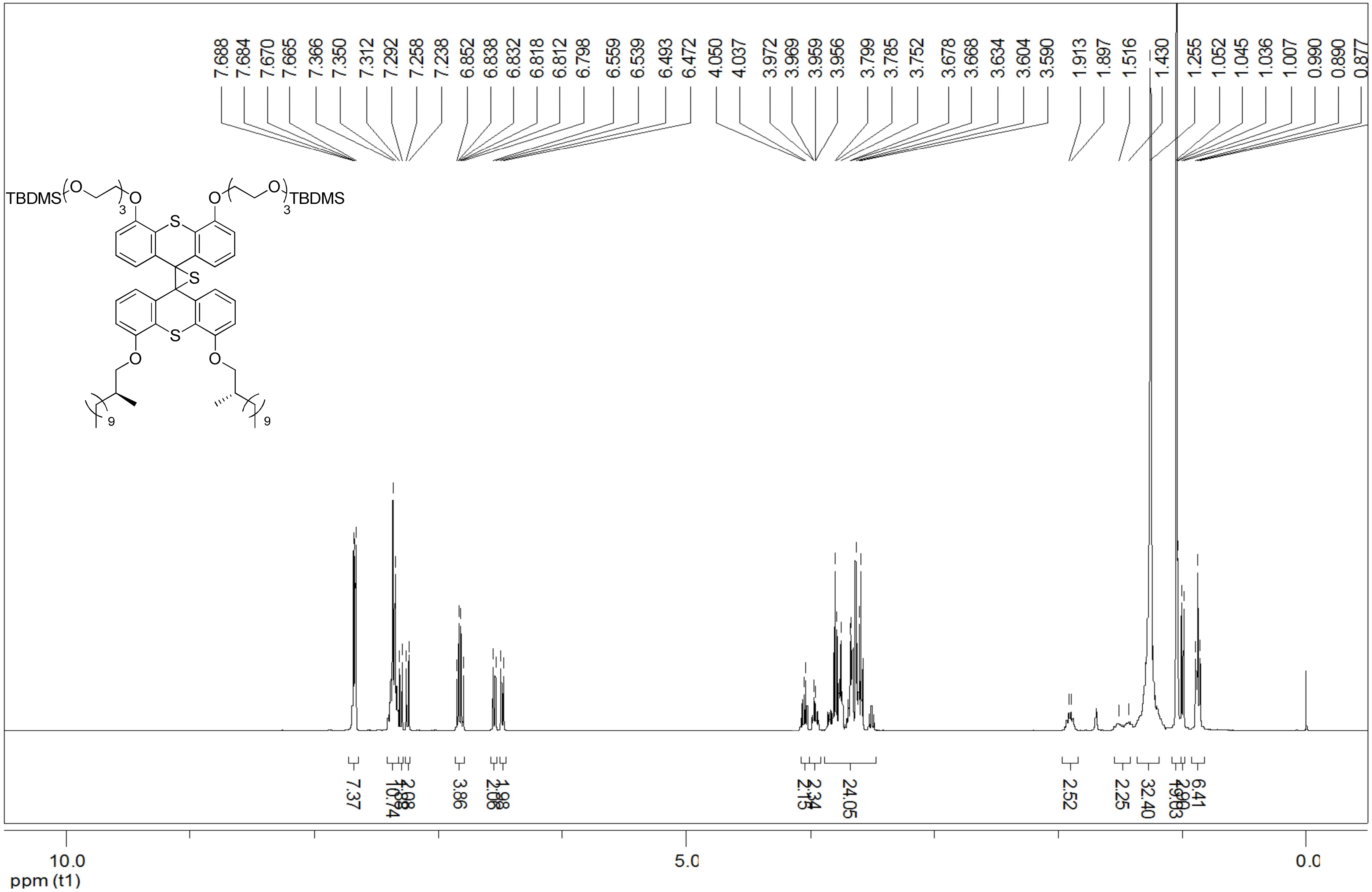

Figure S9. ^{1}H NMR spectrum of **22** in $CDCl_3$ at 400 MHz.

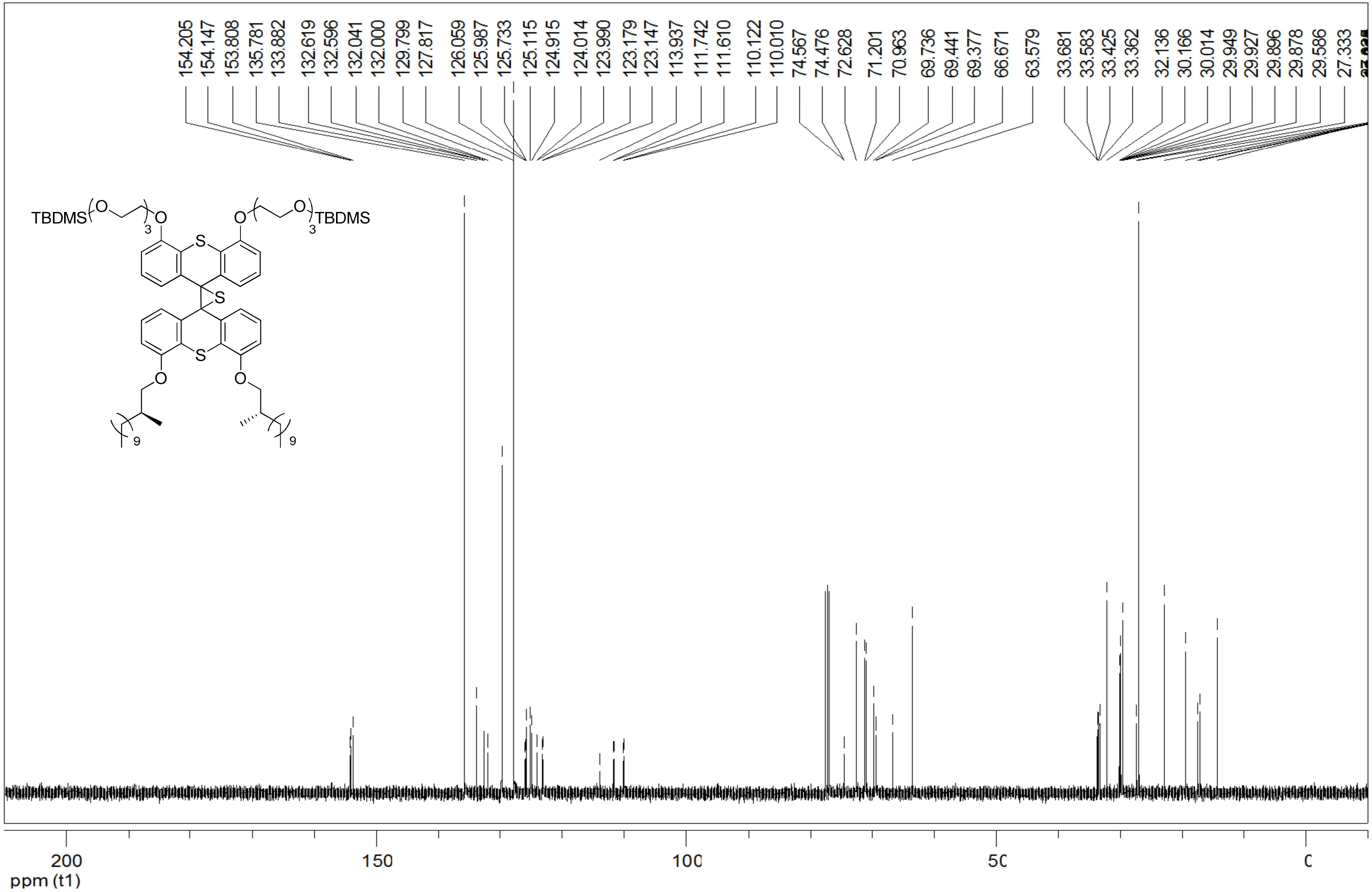

Figure S10. ^{13}C NMR spectrum of **22** in $CDCl_3$ at 100.6 MHz.

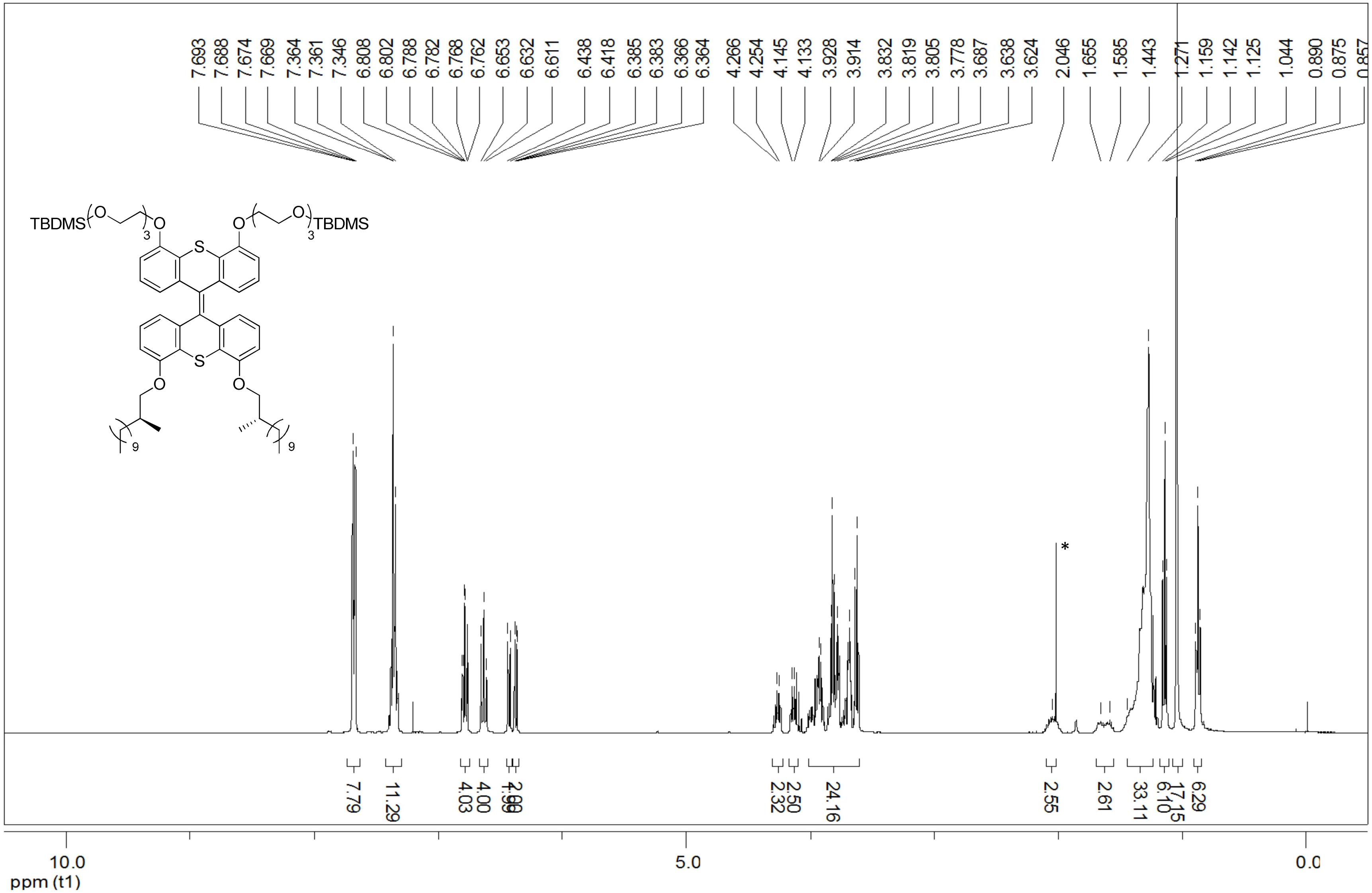

Figure S11. ^{1}H NMR spectrum of **23** in $CDCl_3$ at 400 MHz. *acetone

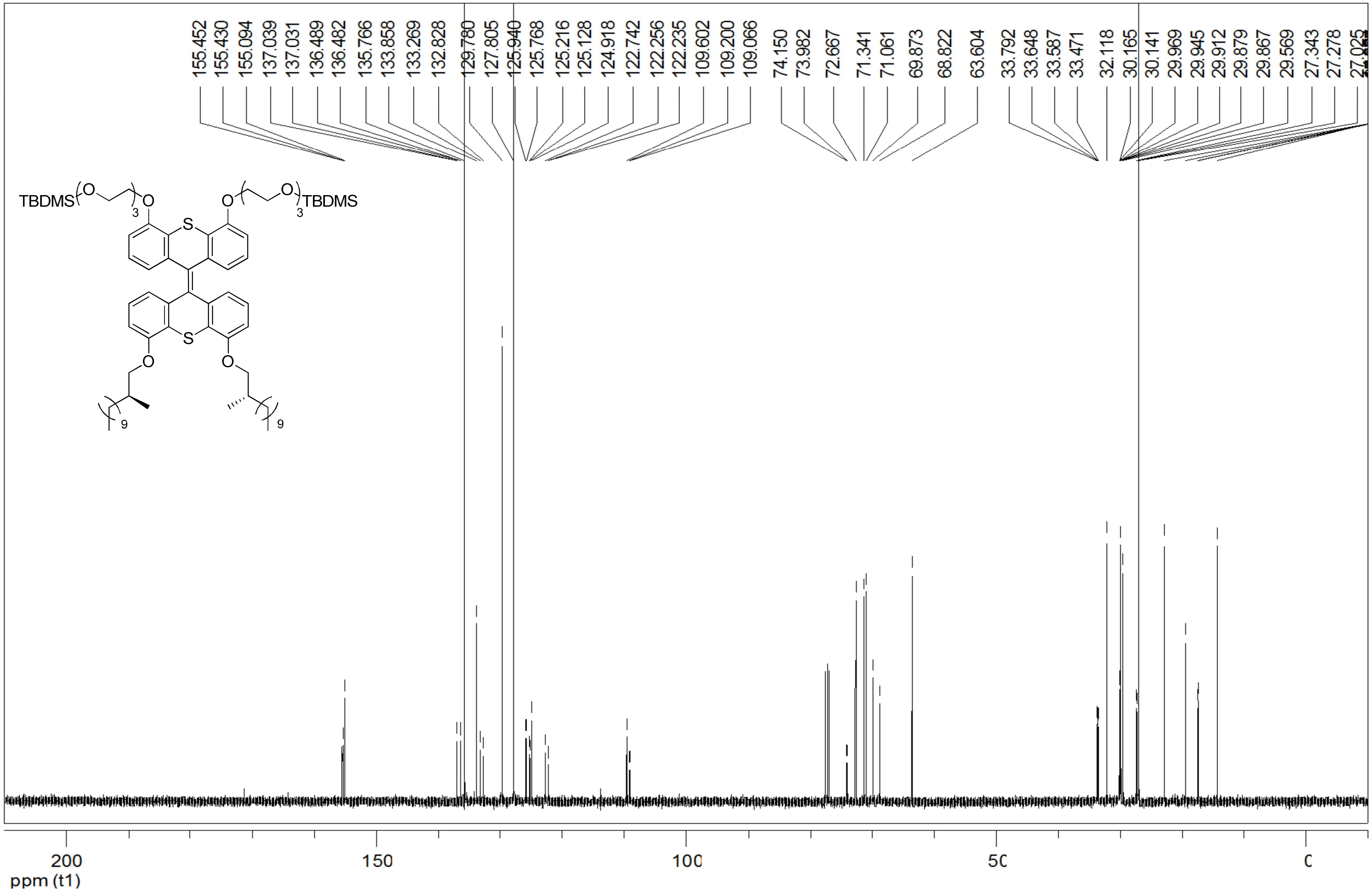

Figure S12. ^{13}C NMR spectrum of **23** in $CDCl_3$ at 100.6 MHz.

3 Self-assembly experiments

For the self-assembly experiments, the compounds (typically 1.0 mg) were weighed on an analytical balance (Mettler MT5 analytical microbalance) and dissolved in chloroform to give a 1 mg/mL stock solution. The stock solutions were never stored for more than a day and typically used immediately after preparation. Compounds **1**, **2** and DOPC were then mixed from the chloroform stock solutions in the desired ratio in a 2 mL glass vial and the mixture (typically 200 – 400 µL total volume) was vortexed for a few min. The mixture was subsequently vacuum-dried under reduced pressure, while rotating the vial and after all chloroform evaporated, the vial was dried further for 1 h under vacuum. The dried thin film was then rehydrated in H_2O to give a suspension (1 mg/mL). The suspension was subjected to five freeze-thaw cycles in liquid nitrogen and a warm water bath (typically 40 °C), respectively, so that a homogeneous turbid solution was formed. During thawing, the samples were vortexed to stimulate homogenization.

4 Cryo-transmission electron microscopy

For analysis by cryo-transmission electron microscopy (cryo-TEM), the turbid solution (2.5 µL) was placed on a glow-discharged holy carbon coated grid (Quantifoil 3.5/1, QUANTIFOIL Micro Tools GmbH, Großlöbichau, Germany). After blotting at room temperature, the grid was rapidly frozen in liquid ethane (Vitrobot, FEI, Eindhoven, The Netherlands) and stored in liquid nitrogen until observed. Grids were observed in a Gatan model 626 cryo-stage in a Philips CM120 cryo-electron microscope operating at 120 KeV. Images were recorded under low-dose conditions on a slow-scan CCD camera.

4.1 Enlarged cryo-TEM images

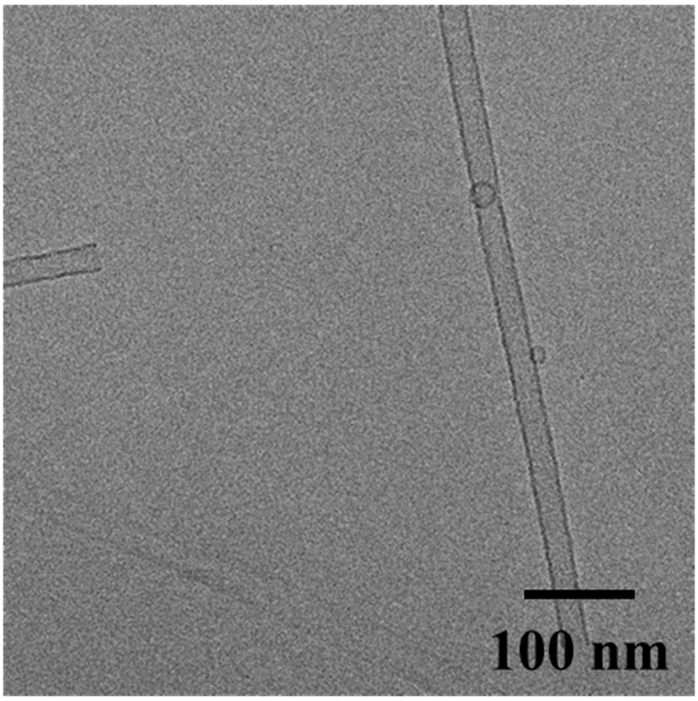

Figure S13 (**Figure 2a,** main text). Cryo-TEM microscopy image of self-assembled nanotubes of achiral **1** with DOPC (1:1) in water at a total concentration of 1 mg/mL.

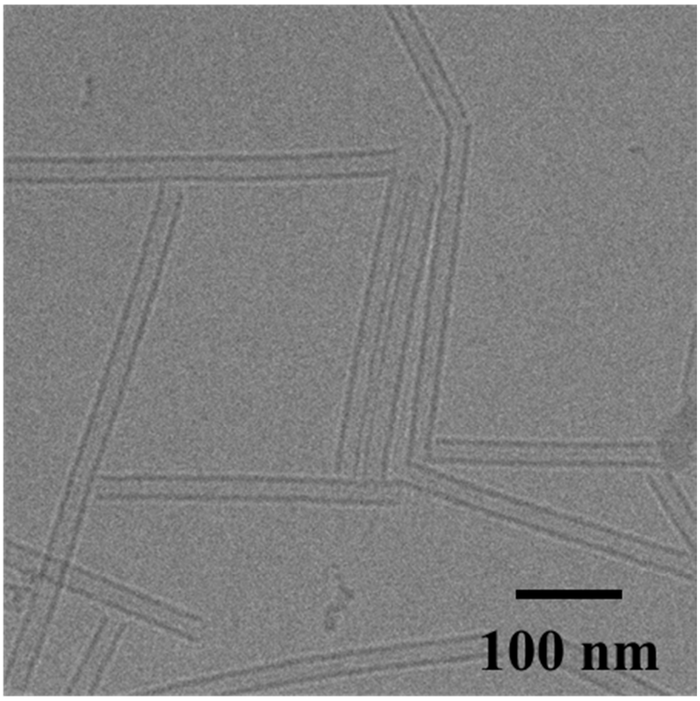

Figure S14 (**Figure 2b,** main text). Cryo-TEM microscopy image of self-assembled nanotubes of chiral **2** with DOPC (1:1) in water at a total concentration of 1 mg/mL.

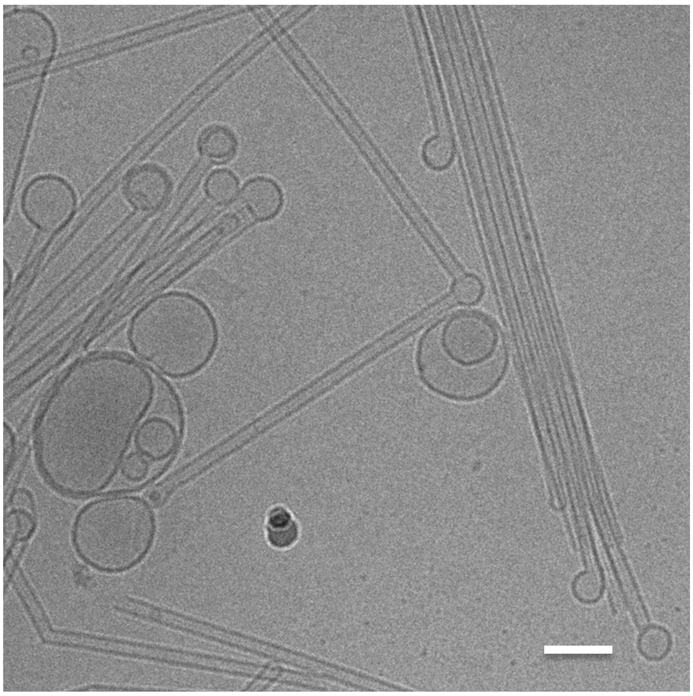

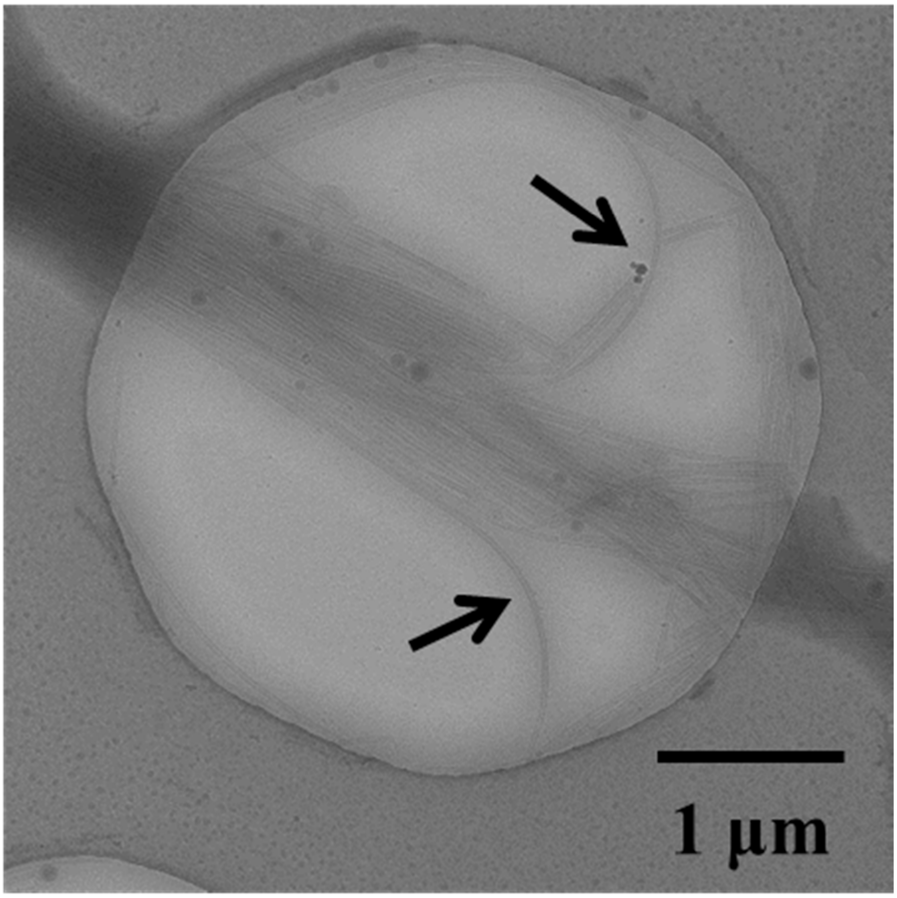

Figure S15 (**Figure 2c,** main text). Cryo-TEM microscopy image of self-assembled nanotubes of **1** and **2** with DOPC (0.6:0.4:1) in water at a total concentration of 1 mg/mL. Arrows indicate nanotubes bending away from the “bundle”.

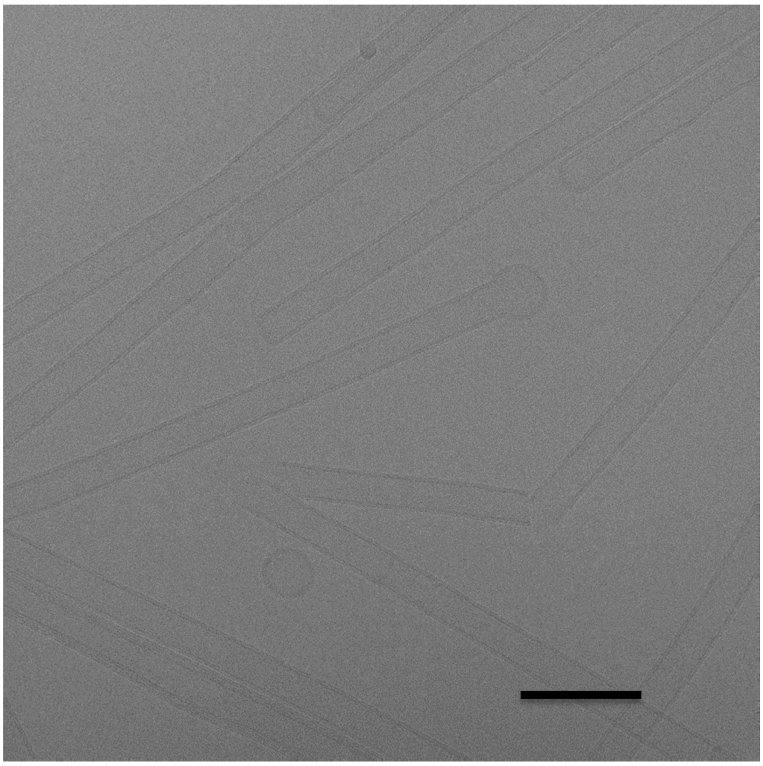

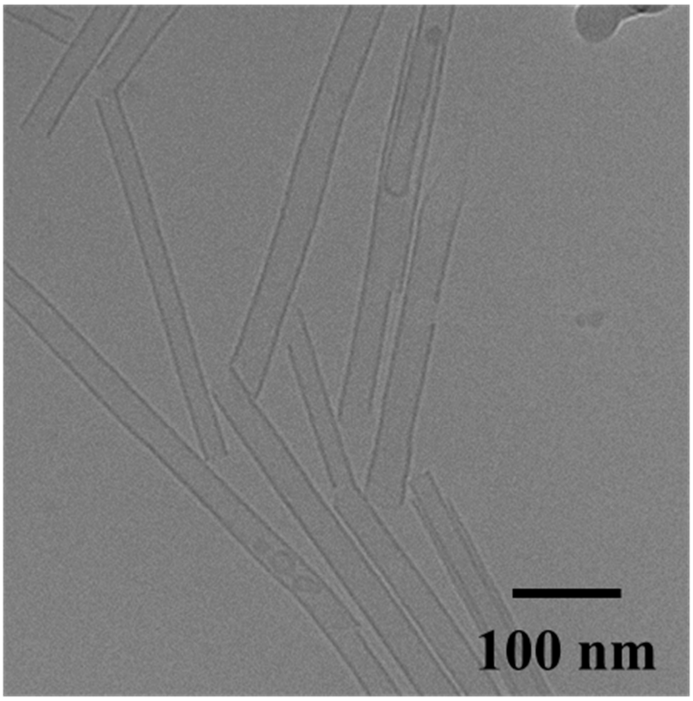

Figure S16 (**Figure 2d,** main text). Cryo-TEM microscopy image of self-assembled nanotubes of **1** and **2** with DOPC (0.4:0.6:1) in water at a total concentration of 1 mg/mL.

5 UV-vis spectroscopy

The absorption properties of the different nanotubes were studied using UV-vis spectroscopy (Figure S18). For UV-vis measurements, the samples were diluted after treated as described in the main text (e.g. after irradiation of the concentrated sample).

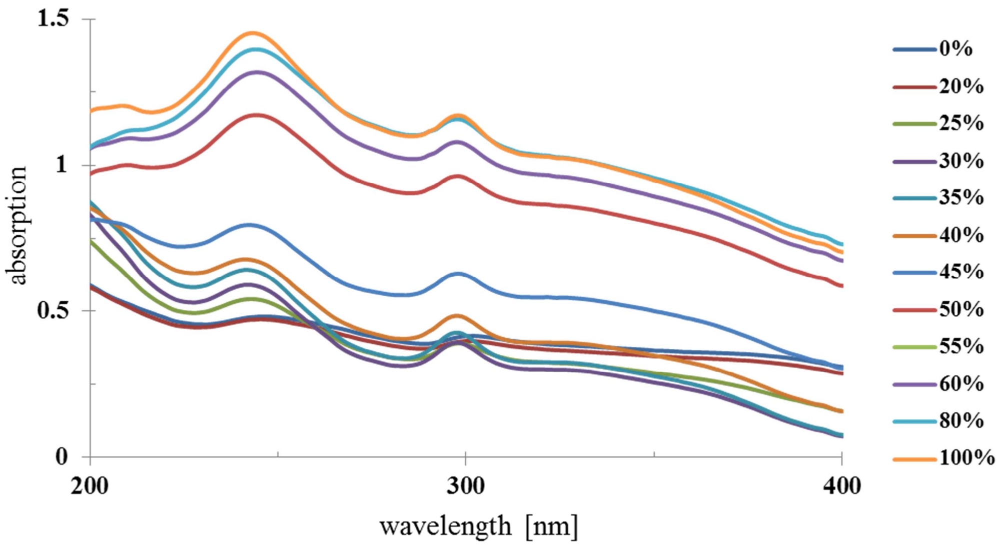

Figure S18. UV-vis absorption spectra of nanotubes in water, having different ratios of **1** and **2**, expressed as a percentage of **2**. The concentration of all samples is $1.6 \cdot 10^{-4}$ M and the total amphiphile to DOPC ratio is 1:1.

6 Light-induced disassembly

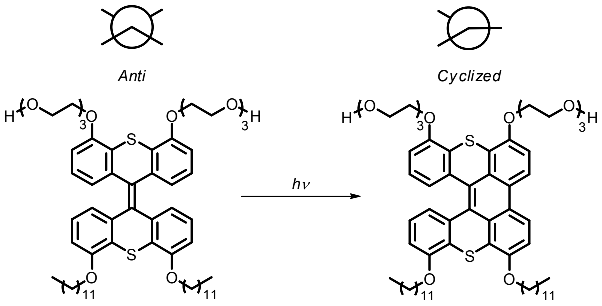

Figure S19. Antifolded structure of photoresponsive amphiphiles, exemplified for **1**, prior to and cyclized form of **1** after irradiation with UV light.

The cyclized form of **1** and **2** is less flexible than the antifolded, uncyclized form and the nanotubes are believed to pack in such a tight manner, that loss of flexibility breaks up the tubes. Indeed, pure cyclized (before self-assembly) **1** formed vesicles upon self-assembly in water,[1] rather than nanotubes, both in the presence and absence of DOPC, providing further evidence for the hypothesis that loss of flexibility, or a change in structure, leads to disassembly of the nanotubes.

6.1 Widfield fluorescence microscopy

Wide field images were obtained using 60x oil immersion objective a Nikkon Ecllipse-Ti with an ANDOR EMCCD-Ixon-DU888 camera and an Andor laser combiner and AOTF unit for illumination at 488 nm.

We followed the disassembly of the different nanotubes in real time, using widefield fluorescence microscopy (Figure S20). The samples were used as described above (self-assembly experiments) for analysis by widefield fluorescence spectroscopy.

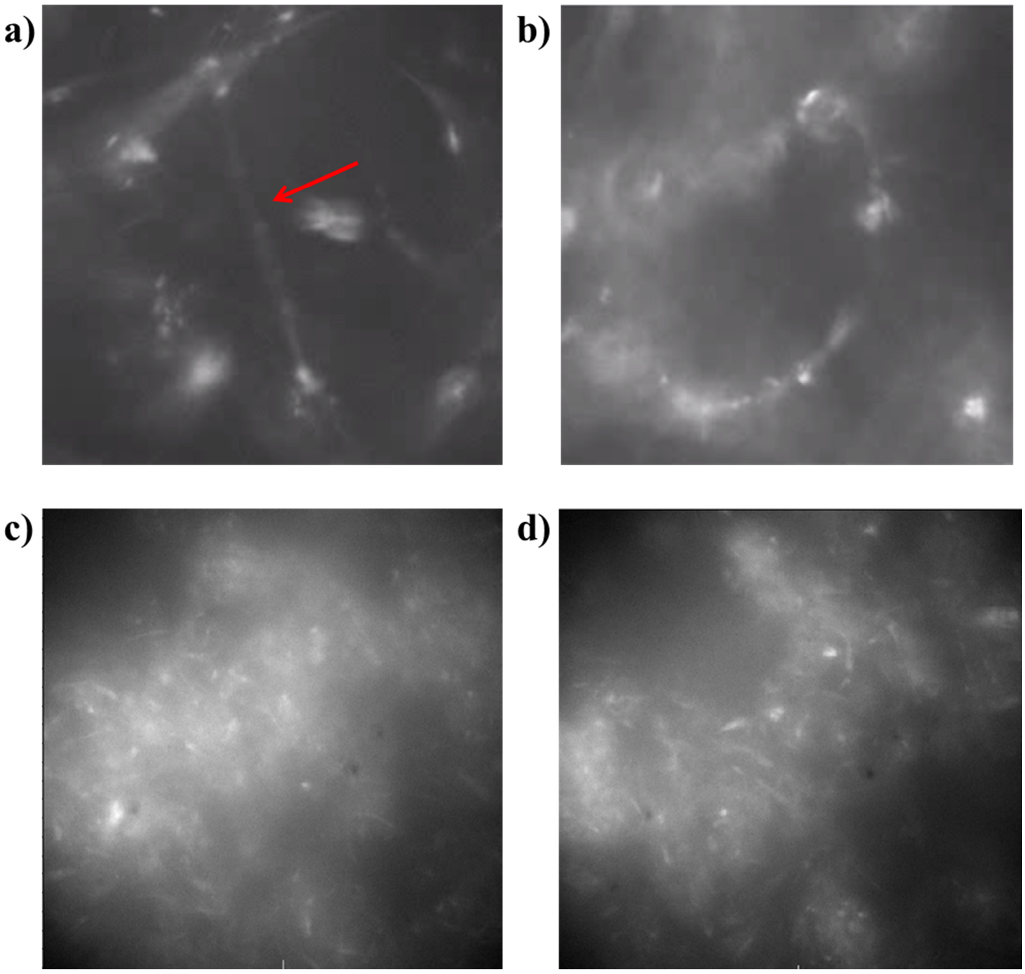

Figure S20. Widefield fluorescence microscopy images of self-assembled nanotubes in water at a total concentration of 1 mg/mL. a) Image of **1** with DOPC (1:1) before and b) after irradiation ($t = 53$ s, $\lambda_{irr} = 390$ nm). c) Image of **2** with DOPC (1:1) before and d) after irradiation ($t = 53$ s, $\lambda_{irr} = 390$ nm).

As shown above, irradiation of the nanotubes of **1** leads to photochemical ring closure of the amphiphile and consequent disassembly of the nanotubes.[1,7] To our delight, upon irradiation, both long tubes of pure **1** (Figure S20a-b), short, clustered tubes of **2** (Figure S20c-d) and mixtures thereof (data not shown), disassemble and large, less defined aggregates are formed. For the short, bundled nanotubes, networks were observed and changes in morphology upon irradiation are therefore less clear, although reproducible and well-pronounced. This finding shows that by changing the chiral content of the nanotubes, the morphology changes, without affecting the photoresponsiveness of the tubular assemblies.

6.2 CD spectroscopy

Subsequently, the loss of CD signal upon low intensity UV irradiation was followed in time for mixed nanotube (**1** + **2**) samples consisting of 35% **2** (long chiral nanotubes) and 55% **2** (short chiral nanotubes) and is shown in Figure S21. For CD measurements, the samples were diluted after treated as described in the main text (e.g. after irradiation of the concentrated sample).

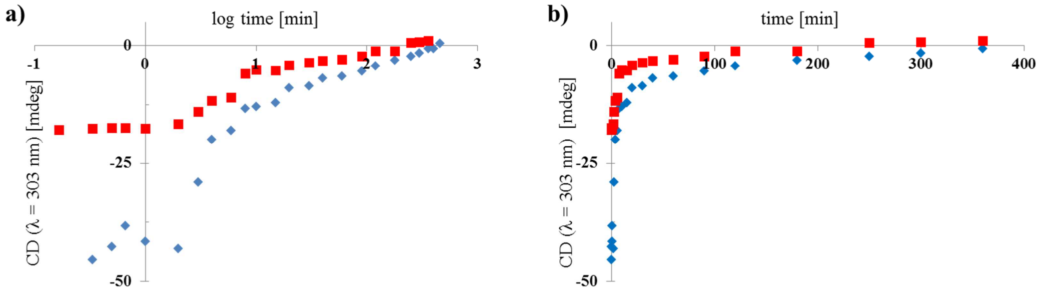

Figure S21. Low intensity UV irradiation (λ_{irr} = 265 nm, 8 W) of long chiral nanotubes consisting of 35% **2** (blue diamonds, $1.6{\cdot}10^{-4}$ M in water) and short chiral nanotubes consisting of 55% **2** (red squares, $1.6{\cdot}10^{-4}$ M in water), followed in time by CD spectroscopy (λ = 303 nm). The time is plotted on a) logarithmic scale and on b) linear scale.

Both long and short chiral nanotubes show a lag period for disassembly under the given conditions of 2 min. We hypothesize that upon initial irradiation, very little molecules are cyclized and disassembly of the nanotubes initiates after a certain threshold is reached. After the lag period, initial disassembly is relatively fast and slows down in time. After approximately 6-8 h, the samples are CD silent, the chiral information in the system erased due to disassembly of the tubes.

7 References

[1] A. C. Coleman, J. M. Beierle, M. C. A. Stuart, B. Macia, G. Caroli, J. T. Mika, D. J. van Dijken, J. Chen, W. R. Browne and B. L. Feringa, *Nat. Nanotechnol.* 2011, **6**, 547.
[2] M. M. Pollard, M. K. ter Wiel, R. A. van Delden, J. Vicario, N. Koumura, C. R. van den Brom, A. Meetsma and B. L. Feringa, *Chem. - Eur. J.* 2008, **14**, 11610.
[3] C. Hermann, C. Giammasi, A. Geyer and M. E. Maier, *Tetrahedron* 2001, **57**, 8999.
[4] T. Sumiyoshi, K. Nishimura, M. Nakano, T. Handa, Y. Miwa and K. Tomioka, *J. Am. Chem. Soc.* 2003, **125**, 12137.
[5] T. Wakabayashi, K. Moriand S. Kobayashi, *J. Am. Chem. Soc.* 2001, **123**, 1372.
[6] P. A. Levene and L. A. Mikeska, *J. Biol. Chem.* **1929**, **84**, 571.
[7] A. C. Coleman, J. Areephong, J. Vicario, A. Meetsma, W. R. Browne and B. L. Feringa, *Angew. Chem. Int. Ed.* 2010, **49**, 6580.
